# The efficacy and safety of Dachaihu decoction in the treatment of nonalcoholic fatty liver disease: a systematic review and meta-analysis

**DOI:** 10.3389/fmed.2024.1397900

**Published:** 2024-07-02

**Authors:** Zhiqing Mou, Tao Gong, Yanzuo Wu, Jun Liu, Jianhua Yu, Lichan Mao

**Affiliations:** ^1^Zhejiang Chinese Medical University, Hangzhou, China; ^2^Hangzhou Xixi Hospital, Hangzhou, China; ^3^Hangzhou Hospital of Traditional Chinese Medicine, Hangzhou, China

**Keywords:** Dachaihu decoction, nonalcoholic fatty liver disease, traditional Chinese medicine, meta-analysis, systematic review

## Abstract

**Background:**

Nonalcoholic fatty liver disease (NAFLD), also known as metabolic associated fatty liver disease (MAFLD), is a common liver condition characterized by excessive fat accumulation in the liver which is not caused by alcohol. The main causes of NAFLD are obesity and insulin resistance. Dachaihu decoction (DCHD), a classic formula in traditional Chinese medicine, has been proved to treat NAFLD by targeting different aspects of pathogenesis and is being progressively used in the treatment of NAFLD. DCHD is commonly applied in a modified form to treat the NAFLD. In light of this, it is imperative to conduct a systematic review and meta-analysis to assess the effectiveness and safety of DCHD in the management of NAFLD. There is a need for a systematic review and meta-analysis to assess the effectiveness and safety of modified DCHD in treating NAFLD.

**Objective:**

The objective of this meta-analysis was to systematically assess the clinical effectiveness and safety of DCHD in treating NAFLD.

**Methods:**

This meta-analysis adhered to the Preferred Reporting Items for Systematic Reviews and Meta-Analyses (PRISMA) guidelines. Including seven databases, both Chinese and English databases were searched for relevant studies. The quality of included studies was carefully assessed using the bias risk assessment tool in the Cochrane Handbook. Eligible articles were the source of extracted data which was meta-analyzed by using Review Manager 5.4 and Stata 17.0.

**Results:**

A total of 10 studies containing 825 patients were included. Compared with conventional treatments, combined treatment could clearly improve the liver function of NAFLD patients, which could reduce the levels of ALT (MD = −7.69 U/L, 95% CI: −11.88 to −3.51, *p* < 0.001), AST (MD = −9.58 U/L, 95% CI: −12.84 to −6.33, *p* < 0.01), and it also had a certain impact on regulating lipid metabolism, which could reduce the levels of TC (MD = −0.85 mmol/L, 95% CI: −1.22 to 0.48, *p* < 0.01), TG (MD = −0.45 mmol/L, 95% CI: −0.64 to 0.21, *p* < 0.01). Adverse event showed that DCHD was relatively safe. Due to the inclusion of less than 10 trials in each group, it was not possible to conduct a thorough analysis of publication bias.

**Conclusion:**

According to the meta-analysis, in the treatment of the NAFLD, it is clear that the combination of DCHD was advantages over conventional treatment alone in improving liver function, regulating lipid metabolism. Additionally, DCHD demonstrates a relatively safe profile. Nevertheless, due to limitations in the quality and quantity of the studies incorporated, the effectiveness and safety of DCHD remain inconclusive. Consequently, further high-quality research is imperative to furnish more substantial evidence supporting the widespread clinical application of DCHD.

**Systematic review registration:**

https://www.crd.york.ac.uk/prospero/display_record.php?ID=CRD42023397353, CRD42023397353.

## 1 Introduction

Nonalcoholic fatty liver disease (NAFLD) is a clinicopathological syndrome characterized by steatosis and fat storage in liver parenchymal cells without a history of excessive alcohol consumption and is commonly associated with metabolic comorbidities such as obesity, diabetes mellitus, and dyslipidemia (1). The vast majority of patients with NAFLD can be asymptomatic and are diagnosed only at physical examination. The pathological process may be hepatic inflammation and hepatic fibrosis, followed by non-alcoholic steatohepatitis (NASH) and NASH can eventually lead to liver cirrhosis, liver failure, and liver cancer (2). In addition, NAFLD can increase the risk of cardiovascular disease and is an independent risk factor for type 2 diabetes (3, 4). The presence and pattern of steatosis along with the degree of inflammation and fibrosis are important pathological characteristics defining the spectrum of NAFLD (5). In recent years, the incidence of NAFLD has been on the rise due to improved living standards and unhealthy eating habits. The global prevalence of NAFLD is estimated to be around 25%, equating to over 1 billion individuals with NAFLD worldwide (6). This not only affects patients’ quality of life, but also imposes a huge medical and economic burden on individuals and society. In the future, as the incidence of hepatitis C decreases, NAFLD is highly likely to be the main form of chronic liver disease in adults and children, and may be the main indication for liver transplantation (7). How to effectively prevent the occurrence, progression of NAFLD and the occurrence of related diseases will be a major public health problem (8). Currently, the main treatment of NAFLD is lifestyle intervention-based, supplemented by medications, with liver transplantation for end stage cases. Some emerging treatments such as anti-inflammatory and antioxidant therapies are also being tested in clinical trials, but more evidences are still needed to support their efficacy (9–11). However, it is hard to comply to a long-term lifestyle intervention (12). The effects of current pharmacological therapies are limited and cannot completely halt the progression of NAFLD, and the adverse effects of some drug treatments are too severe for patients to tolerate (13). Liver transplantation has problems such as donor shortage, postoperative recurrence, and postoperative complications (14). Meanwhile, no therapeutic modality can directly target the progression of liver fibrosis, which is the most critical pathological change affecting the prognosis of NAFLD (15). Therefore, there is an urgent need to discover safer and more effective treatment options.

In recent years, traditional Chinese medicine (TCM) has shown promising potential in the treatment of NAFLD. Research has demonstrated that TCM formulas have multi-target pharmacological effects that can simultaneously regulate hepatic lipid metabolism (16–18), improve insulin sensitivity (17), inhibit inflammatory factors (18, 19) and anti-oxidation (19), thereby treating NAFLD in an integrated manner targeting various aspects of pathogenesis. Some TCM ingredients can inhibit activation of hepatic stellate cells, the key cells in liver fibrosis, improving NAFLD pathological changes in the liver and slowing disease progression (20–24). Compared with western medicine, TCM treatment for NAFLD has the advantages of low incidence of adverse reactions (24), good long-term medication adherence (17) and low economic cost (22). One classical TCM formula used in the treatment of NAFLD is Dachaihu decoction (DCHD). It originated from the ancient Chinese medical text “Treatise on Cold Damage and Miscellaneous Diseases” written by Zhongjing Zhang during the Eastern Han Dynasty. DCHD is composed of Chinese Thorowax Root (Chaihu, *Bupleurum falcatum* L.), Baical Skullcap Root (Huangqin, *Scutellaria baicalensis* Georgi), Rhubarb (Dahuang, *Rheum palmatum* L.), Immature Orange Fruit (Zhishi, *Citrus aurantium* L.), Pinellia Tuber (Banxia, *Pinellia ternata* (Thunb.) Makino), White Paeony Root (Baishao, Paeonia lactiflflora Pall.), Chinese Date (Dazao, *Ziziphus jujuba* Mill.) and Fresh Ginger (Shengjiang, *Zingiber offificinale* Roscoe). DCHD has the effects of harmonizing Shaoyang and purging heat stagnation internally. It is commonly used to treat Shaoyang combined Yangming disease, whose main manifestations are alternating chills and fever, chest and hypochondriac distention and fullness, vexation, vomiting, constipation, etc. The main active components of DCHD measured by high performance liquid chromatography (HPLC) include paeoniflflorin, naringin, hesperidin, neohesperidin, baicalin, baicalein and saikosaponin A (25, 26). These components have been found to possess regulate bile acid metabolism (27), anti-inflammatory properties (27, 28), balance intestinal flora (29, 30), protect liver function (29), and modulate blood lipids (29–32). So DCHD has traditionally been used to treat conditions such as cholecystitis (33), hyperlipidemia (34, 35), bile reflux gastritis (36), and acute pancreatitis (27). Recently, it has also been explored for the treatment of NAFLD. DCHD or its modified forms may help alleviate clinical symptoms associated with NAFLD, such as abdominal distension, abdominal pain, and loss of appetite. However, the clinical efficacy of DCHD in treating NAFLD is still uncertain due to limited sample sizes, variations in efficacy indicators, inconsistent trial designs, and ambiguous methodological quality. Additionally, there is a lack of clinical evidence summarizing the efficacy and safety of DCHD specifically in NAFLD treatment. Therefore, this study aims to comprehensively collect randomized controlled trials (RCTs) evaluating the use of DCHD alone or in combination with conventional treatments for NAFLD. The goal is to evaluate the clinical efficacy and safety of DCHD and provide valuable insights for future research and clinical practice.

## 2 Materials and methods

### 2.1 Study registration

This systematic review and meta-analysis followed the guidelines outlined in the Cochrane Handbook for Systematic Reviews of Interventions version 6.3 (updated 2022) and the 2020 Statement of the Preferred Reporting Items for Systematic Review and Meta-Analysis (PRISMA) (37, 38). Supplementary material S1 includes the PRISMA 2020 checklist used in this study. Prior to conducting the research, the study was registered in the International Prospective Register of Systematic Reviews (PROSPERO) with the registration number CRD42023397353. All data analyzed in this study were obtained from published clinical studies.

### 2.2 Database and search strategies

We had made a comprehensive search of three English electronic databases, including EMBASE, PubMed, and the Cochrane Library, and four Chinese electronic databases, namely the China National Knowledge Infrastructure (CNKI), Wan Fang Database, Weipu Database and Chinese Biomedical Literature Database (CBM) from their inceptions to January 2023. The clinical trials associated with DCHD, modified DCHD, NAFLD were searched using a combination of subject terms and text words. The search terms mainly included: “Daisaikoto,” “Dachaihu,” “Dachaihu Decoction,” “Dachaihu Tang,” “Da Chaihu,” “Da Chaihu Tang,” “Da Chaihu Decoction,” “Major Bupleurum Decoction,” “Major Bupleurum Tang,” “Non alcoholic Fatty Liver Disease,” “NAFLD,” “Nonalcoholic Fatty Liver Disease” and “Nonalcoholic Steatohepatitis.” Additional search terms can be found in Supplementary material S2. Ongoing studies were also identified through the ClinicalTrials.gov database and the Chinese Clinical Trial Registry (CHiCTR). Furthermore, references of relevant meta-analyses and reviews were reviewed to ensure no literature was missed in the online searches. Only original articles in English or Chinese were included, and selection criteria were applied to include literature accordingly.

### 2.3 Inclusion criteria

Type of studies: RCTs with high-quality evidence assessing interventions for NAFLD would be included regardless of their source or country. The publication language would be limited to English or Chinese.

Type of participants: The study population would consist of adults (≥18 years old) diagnosed with NAFLD, without any restrictions based on complications. There would be no demographic restrictions, such as race, age, or gender.

Type of interventions: Interventions involving DCHD or modified DCHD would be included, without limitations on dosage form (capsule, decoction, or granules), frequency, or dosage. The experimental group would receive DCHD alone or DCHD combined with conventional treatment. The control group could receive either placebo or conventional treatment. Conventional treatment refers to the standard treatment approaches of Western medicine or other traditional Chinese medicine. Standard treatment measures of Western medicine may include health education, exercise intervention, diet management, blood lipid monitoring, lipid-lowering drugs. Standard treatment measures of traditional Chinese medicine mainly involve the use of conventional prescriptions, such as Zhishi Daozhi pills. There would be no limitations on the type or dosage form (oral preparation or injection) of traditional Chinese medicine or Western medicine. If the experimental group received combined treatment with conventional treatment, it would be identical to the control group.

Type of comparisons: The following comparisons were made respectively in this study: conventional treatment combined with DCHD vs. conventional treatment; DCHD vs. conventional treatment. There is no analysis in this study regarding the comparison between DCHD and placebo, because no study had compared this aspects.

Type of outcome measures: The evaluation of the efficacy and safety of DCHD for NAFLD was performed by analyzing liver function, lipid metabolism, insulin resistance, glucose metabolism, body mass index (BMI) and adverse events, etc. RCTs that assessed any of these outcomes were included in the study:

1. Primary outcomes

Liver function index: Alanine aminotransferase (ALT), Aspartate aminotransferase (AST).

Lipid metabolism index: Total cholesterol (TC), Triglyceride (TG).

1. Secondary outcomes

Liver function index: γ-glutamyl transpeptadase (γ-GGT).

Lipid metabolism index: High-density lipoprotein cholesterol (HDL-C), Low-density lipoprotein cholesterol (LDL-C).

Body mass index (BMI).

If a study reported multiple time points, only the result from the longest time point was considered for analysis.

1. Safety outcomes

Any adverse events that occurred during the study, such as gastrointestinal adverse reactions, overall adverse event incidence, and serious adverse event incidence, were recorded.

### 2.4 Study selection and data extraction

The search results were imported into EndNote X20 software to establish a database in the form of bibliography. Two researchers independently screened the literature based on inclusion and exclusion criteria. Duplicate literature was removed first, followed by preliminary screening of titles and abstracts for literature that did not meet the criteria. Literature that remained uncertain after preliminary screening was reviewed in full text. After reading the full text, literature that still did not meet the criteria was excluded. Any discrepancies were resolved through discussion or consultation with LM and JY. Data extraction from included studies was conducted independently by two researchers using a pre-designed data extraction table. If additional data were needed, the authors were contacted via email. The extracted research data mainly included the first author, publication year, study design, gender, sample size, average age, duration of disease, treatment duration, intervention measures, outcome indicators, baseline differences, comorbidity, adverse events, diagnostic criteria, country, funding, and was crosschecked.

### 2.5 Risk of bias assessment

The quality of the included literature was assessed using the bias risk assessment tool from the Cochrane Handbook. This assessment was conducted within the Review Manager 5.4 software. The tool consisted of seven important sources of bias, including random sequence generation, allocation concealment, blinding of participants and personnel, blinding of outcome assessment, incomplete outcome data, selective reporting, and other biases. Each study was evaluated for the risk of bias in these seven aspects. After accessing the completeness of research reporting and the correctness of methodological implementation, each aspect was assessed as “high risk,” “low risk” or “unclear risk.” Two independent researchers reviewed and cross-checked each other’s assessments. In case of any discrepancies, a third researcher participated in the discussion to reach a final decision.

### 2.6 Statistical analysis

Meta-analysis was performed using Review Manager 5.4 and Stata 17.0 software. For continuous variables with the same outcome indicator and unit, the mean difference (MD) and 95% confidence interval (CI) were used as the effect size. Otherwise, the standardized mean difference (SMD) and 95% CI were utilized. For binary variables, the relative risk (RR) and 95% CI were employed. Heterogeneity was assessed using the *χ*^2^ test and *I*^2^ test. If *p* > 0.1 and *I*^2^ < 50%, it indicated low heterogeneity among the studies, and the fixed-effect model was used to calculate the pooled effect size. If *p* ≤ 0.1 and *I*^2^ ≥ 50%, it suggested significant statistical heterogeneity, and the random-effects model was applied. Subgroup analysis and sensitivity analysis were conducted to explore potential sources of heterogeneity and assess the stability of the results. Meta-regression was performed on sample size, publication year, average age, and the proportion of males for ALT, AST, TC, and TG, as these indicators were included in more studies available. Additionally, funnel plots, Egger’s test, and Begg’s test were used to evaluate publication bias when more than 10 studies were included in the meta-analysis.

### 2.7 Subgroup analysis

We conducted subgroup analyses based on prespecified factors that could potentially impact treatment outcomes in order to explore sources of heterogeneity. The subgroup analyses were performed according to the following criteria: duration of disease (≤2 years or >2 years), treatment duration (≤2 months or >2 months), average age (≤45 years old or >45 years old), presence of comorbidity (yes or no), and type of treatment (traditional Chinese medicine or western medicine).

## 3 Results

### 3.1 Database search results

**Figure 1:**
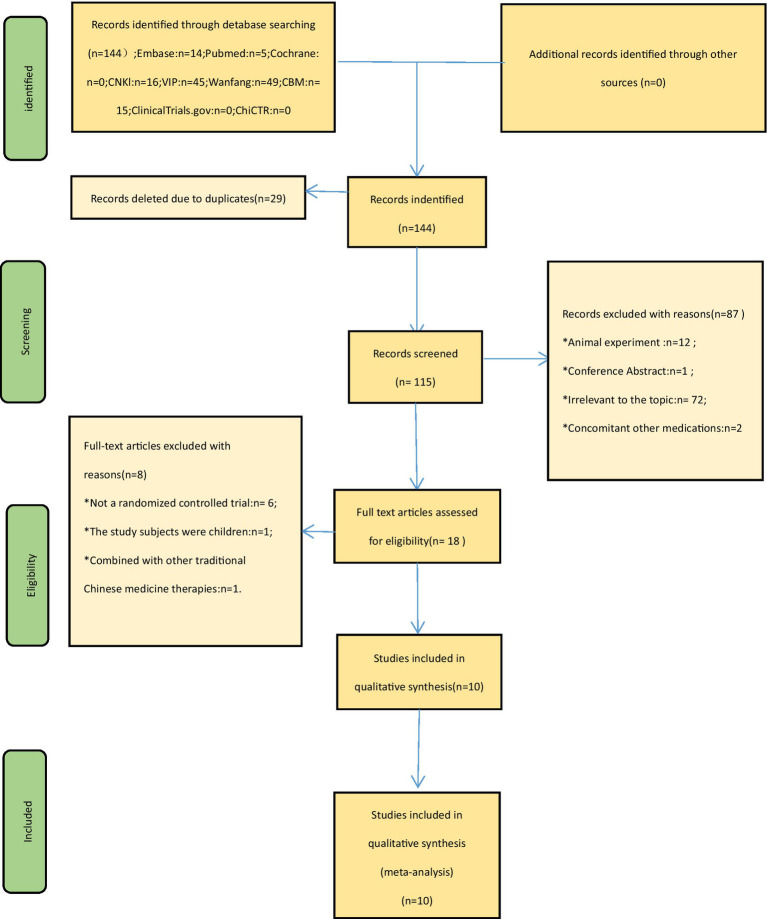
Flowchart of study selection and identification.

A comprehensive search of both Chinese and English databases yielded a total of 144 studies. After removing duplicates, 115 studies remained. Among these, 87 studies were excluded based on title and abstract screening. The full texts of the remaining 18 studies were assessed, and 8 studies were excluded based on the predetermined inclusion and exclusion criteria. Additionally, a thorough review of relevant meta-analyses and reviews led us to a study titled “Meta-analysis of randomized controlled trials on fatty liver treatment.” However, upon careful examination, it was determined that this study did not contain the same content as the present article. Ultimately, 10 eligible studies were included in the quantitative analysis. Please refer to Figure 1 for a detailed flowchart illustrating the process of screening eligible studies.

### 3.2 Characteristics of included study

This study included a total of 10 RCTs conducted in China between 2012 and 2022 (39–48). The study population consisted of 825 patients with NAFLD, with 412 patients in the experimental group and 413 patients in the control group. Eight studies (40–43, 45–48) were treated with modified DCHD, one study (39) was treated with Dachaihu granules and one study (44) was treated with the original DCHD. Through consulting relevant information, it was found that Dachaihu granules had the same herbs as the original DCHD. The composition of DCHD or modified DCHD is detailed in Supplementary material S3, but none of the included studies reported quality control or chemical analysis of DCHD. Seven studies (42–48) had a control group receiving conventional treatment while the experimental group received DCHD or modified DCHD in addition to conventional treatment. The conventional treatment in the control group was consistent with that used in the experimental group. In the remaining 3 studies (39–41), the control group received conventional treatment alone while the treatment group received DCHD or modified DCHD alone. Hence, none of the studies mentioned above were adequately blinded. The basic characteristics of the included studies are summarized in Table 1.

**Table 1:** The characteristics of the included studies.

First author (year)	Jiang et al. (2012)	Shen et al. (2012)	Guan et al. (2013)	Lin et al. (2017)	Zhang (2019)
Study design	RCT	RCT	RCT	RCT	RCT
Sample size (randomized/analyzed) (E/C)	76/76; 36/40	60/60; 30/30	64/64; 32/32	80/80; 40/40	98/98; 49/49
Gender (M/F) (E/C)	42/34 (total)	17/13; 16/14	18/14; 19/13	21/19; 20/20	27/22; 25/24
Average age (years) (E/C)	48.2 ± 10.6 (total)	44.26 ± 6.15; 45.68 ± 6.63	56.94 ± 5.28; 57.06 ± 4.04	43.73 ± 20.10; 42.70 ± 21.20	47.51 ± 6.24; 47.98 ± 6.47
Course of disease (years) (E/C)	NR	3–9 years; 2–9 years	4.21 ± 0.56; 4.15 ± 0.59	NR	1.62 ± 0.68
Treatment duration	6 months	3 months	12 weeks	2 months	3 months
Comorbidity	In the treatment group, 24 cases accompanied with simple obesity and in the control group, 23 cases accompanied by simple obesity	NR	Type 2 diabetes mellitus (T2DM)	NR	NR
Co-intervention	The patients with simple obesity in the treatment group and the control group were treated with diet control and exercise therapy	Low fat diet, appropriately increase the amount of exercise	Reduce blood sugar, blood pressure and fat	Improve diet structure, carry out moderate aerobic exercise and control weight	Protect liver, strengthen aerobic exercise, low salt and low fat diet, etc.
Treatment group interventions	Dachaihu granules 8 g, TID	Modified DCHD, 1 dose/per day, BID	Modified DCHD, 1 dose/per day, BID	Modified DCHD, 1 dose/per day, 400 mL, BID + CG	Modified DCHD, 1 dose/per day, 400 mL, BID + CG
Control group interventions	*Oenothera Biennis* Oil Soft Capsules 1.5 g, BID	Ursodeoxycholic acid 250 mg, TID	Melbine 500 mg, TID	Polyene phosphatidylcholine 456 mg, TID	Zhishi Xiaopi pill, 1 dose/per day, BID
Outcome index	① ② ③ ④	① ② ③ ④ ⑤	③	① ② ④	① ② ③ ⑥ ⑦
Baseline difference	NSD	NSD	NSD	NSD	NSD
Adverse events	NR	NR	NR	Control group: 1 case, details unknown	NR
Diagnostic criteria	2003 CMA	2006 CMA	1999 WHO and 2010 CMA	2010 CMA	NR
Country	China	China	China	China	China
Funding	NR	NR	NR	NR	NR

First author (year)	Bao et al. (2020)	Wang et al. (2020)	Ren (2020)	Zhang (2021)	Xv et al. (2022)
Study design	RCT	RCT	RCT	RCT	RCT
Sample size (randomized/analyzed) (E/C)	60/60; 30/30	157/157; 79/78	92/92; 46/46	80/80; 40/40	58/58; 30/28
Gender (M/F) (E/C)	19/11; 18/12	47/32; 44/34	31/15; 33/13	30/10; 28/12	20/10; 17/11
Average age (years) (E/C)	44.23 ± 12.80; 40.18 ± 11.79	43.01 ± 13.04; 42.92 ± 12.97	42.87 ± 4.03; 43.18 ± 3.91	52.28 ± 1.49; 52.32 ± 1.63	43.15 ± 8.36; 45.58 ± 7.21
Course of disease (years) (E/C)	10.00 ± 14.08; 8.90 ± 14.29 (months)	NR	2.44 ± 0.38; 2.39 ± 0.40	4.48 ± 1.36; 4.52 ± 1.36 (months)	5.93 ± 1.91; 5.73 ± 1.83
Treatment duration	12 weeks	3 months	4 weeks	NR	12 weeks
Comorbidity	Type 2 diabetes mellitus (T2DM)	NR	NR	NR	In the treatment group, 21 cases accompanied with diabetes and 7 cases accompanied by hypertension. In the control group, 18 cases were accompanied by diabetes and 6 cases were accompanied by hypertension
Co-intervention	Diet and exercise intervention	Diet, quit drinking and exercise intervention	High vitamin diet and Aerobic exercise	Aerobic exercise, adequate rest and vitamin supplements in diet	High vitamin diet and Aerobic exercise
Treatment group interventions	DCHD, 1 dose/per day, 400 mL, BID + CG	Modified DCHD, 1 dose/per day, 300 mL, BID + CG	Modified DCHD, 1 dose/per day, 400 mL, BID + CG	Modified DCHD, 1 dose/per day, BID + CG	Modified DCHD, 1 dose/per day, 400 mL, BID + CG
Control group interventions	Exenatide, 5 μg, BID, subcutaneous injection; exenatide, 10 μg, BID, subcutaneous injection (four weeks later)	Silybin 105 mg, BID	Zhishi Xiaopi pill 6 g, TID	Zhishi Xiaopi pill 6 g, BID	Fenofibrate 200 mg, qd + Compound Glycyrrhizin Tablets 100 mg, TID
Outcome index	① ② ③ ④ ⑤ ⑧	① ②	① ② ③ ④	① ② ③ ④	① ④ ⑤
Baseline difference	NSD	NSD	NSD	NSD	NSD
Adverse events	NR	NR	NR	NR	NR
Diagnostic criteria	2010CMA	2017CMA	2011CMA	NR	2017CMA
Country	China	China	China	China	China
Funding	NR	NR	NR	NR	NR

RCT, randomized controlled trial; CHS, Chinese Hepatology Society; NR, not reported; DCHD, Dachaihu decoction; CG, control group interventions; NSD, no significant different. Outcome index:① ALT ② AST ③ TC ④ TG ⑤ γ-GGT ⑥ HDL-C ⑦ LDL-C ⑧ BMI.

### 3.3 Risk of bias assessment

**Figure 2:**
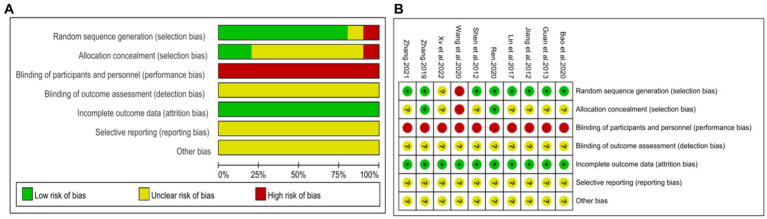
Risk of bias assessment for included studies. **(A)** Risk of bias graph. **(B)** Risk of bias summary.

The Cochrane Risk of Bias tool was used to assess the risk of bias in the included studies. Two studies (43, 46) utilized a random number table and were considered low risk. Five studies (39–42, 44) claimed to have performed randomization but did not provide specific details about the methods used for random sequence generation, resulting in an unclear risk rating. One study (46) employed the odd-even method for allocation, but the adequacy of concealment was not adequately described. Another study (48) did not mention the assignment concealment method. As a result, these seven (39–42, 44, 47, 48) studies were marked as unclear risk. One study (41) grouped participants based on treatment method without adhering to the principles of randomization, resulting in a high risk rating. In these studies, the control group received conventional treatment while the experimental group received DCHD alone or in combination with conventional treatment. However, there were variations in the form, frequency, and dosage of the drug between the experimental and control groups, making it difficult to achieve complete blinding for both participants and researchers. Therefore, these studies were there were variations in the form, frequency, and dosage of the drug between the experimental and control groups, making it difficult to achieve complete blinding for both participants and researchers. Therefore, these studies were rated as high risk in terms of blinding. None of the studies provided information on whether outcome assessors were blinded, resulting in an unclear risk rating for this aspect. All studies had complete data, so all had been labeled as low risk in this aspect. Due to the lack of registration and study protocols, it was not possible to assess the risk of selective reporting. Additionally, there was insufficient information to determine the presence of other significant biases, resulting in an unclear risk rating for all studies. In summary, the methodological quality of the included studies was not high. The risk of bias assessment results for the included studies are presented in Figure 2.

### 3.4 Primary outcomes

#### 3.4.1 ALT

##### 3.4.1.1 Conventional treatment combined with DCHD vs. conventional treatment

**Figure 3:**
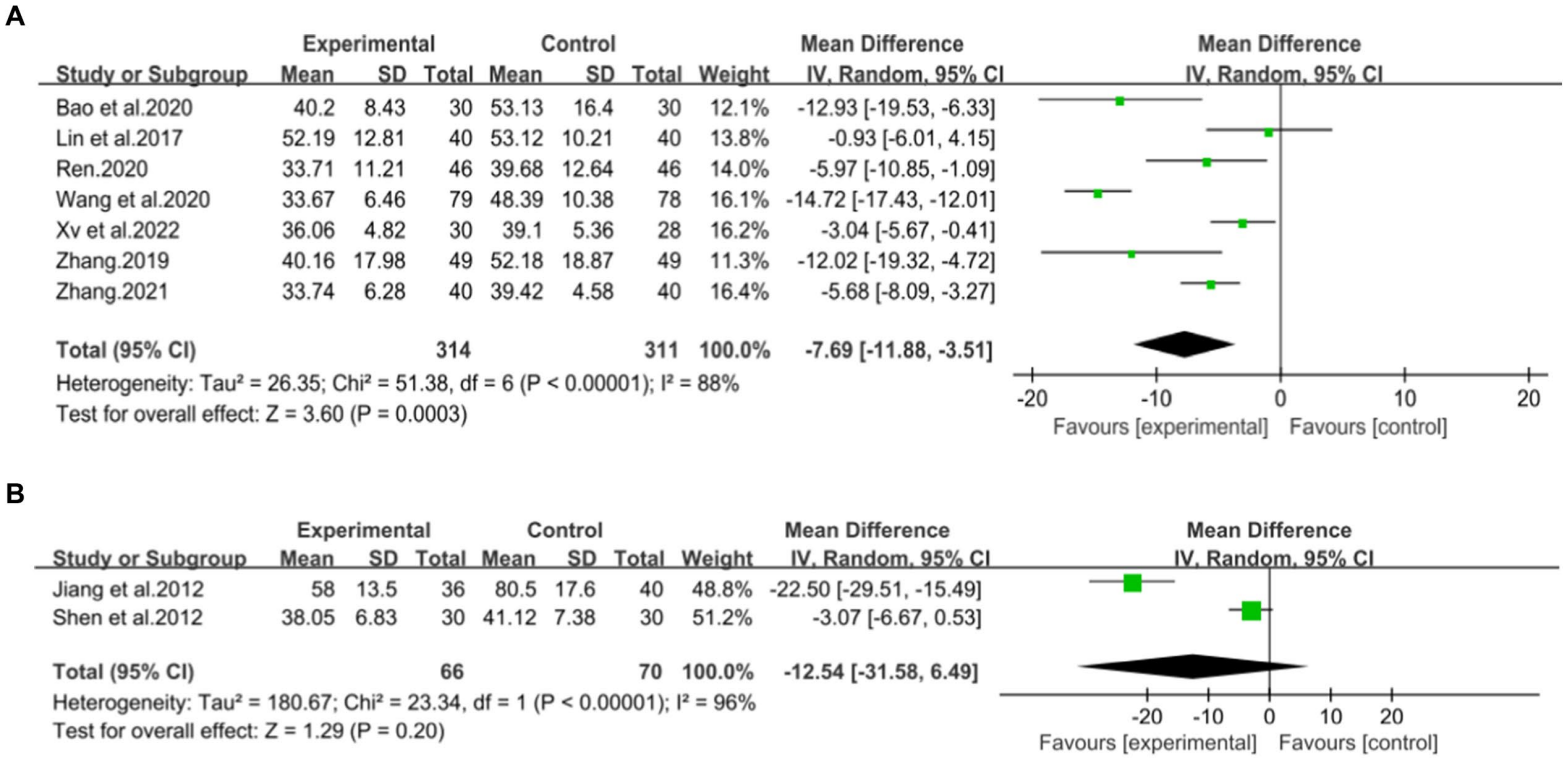
Forest plot of the ALT. **(A)** DCHD combined with conventional treatment vs. conventional treatment. **(B)** DCHD vs. conventional treatment.

**Figure 4:**
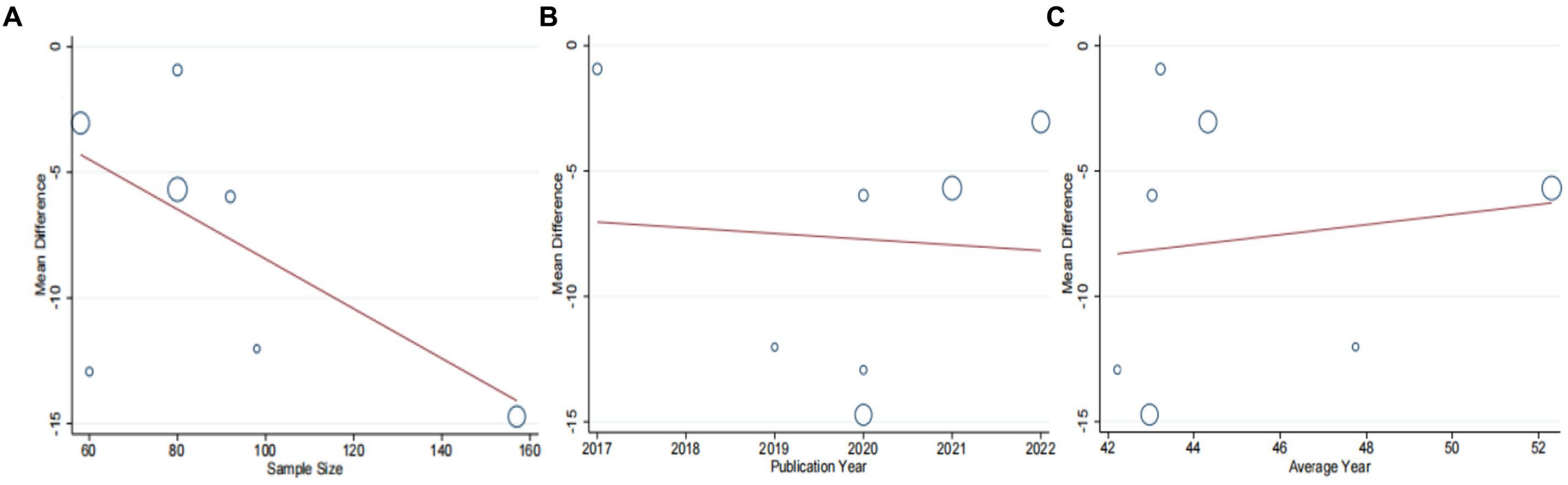
Meta-regression of the ALT for DCHD combined with conventional treatment vs. conventional treatment. **(A)** Sample size. **(B)** Publication year. **(C)** Average year.

**Figure 5:**
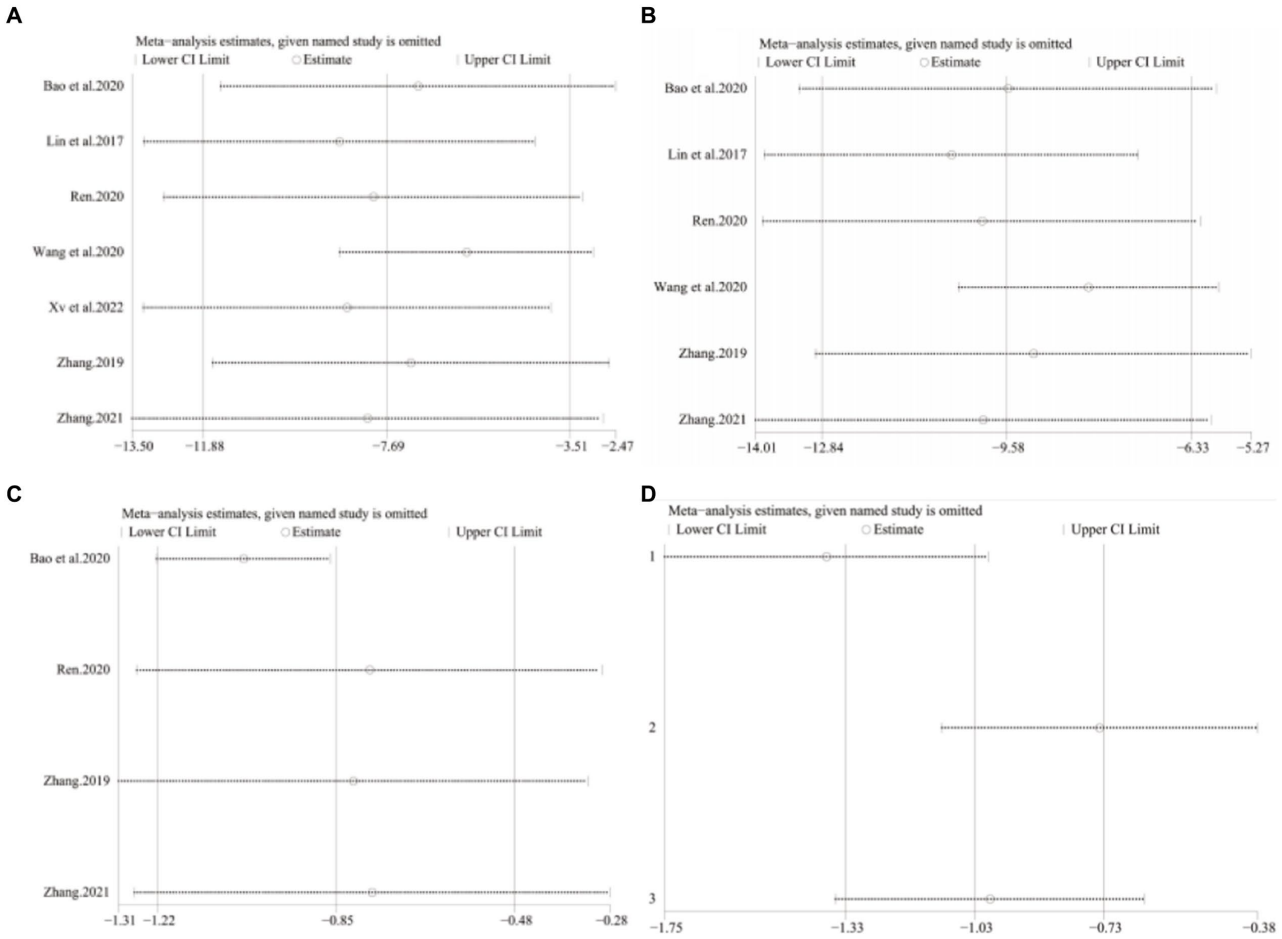
Sensitivity analysis. DCHD combined with conventional treatment vs. Conventional treatment. **(A)** ALT. **(B)** AST. **(C)** TC. DCHD vs. conventional treatment: **(D)** TC.

A total of seven studies (42–48) involving 625 patients with NAFLD, reported on the efficacy of combining conventional treatment with DCHD compared to conventional treatment alone in terms of ALT levels. The heterogeneity test (*p* < 0.01, *I*^2^ = 88%) indicated significant heterogeneity among the studies, therefore a random effect model was used for statistical analysis. The pooled effect demonstrated that the combination of DCHD and conventional treatment resulted in a statistically significant reduction in ALT levels compared to conventional treatment alone (MD = −7.69 U/L, 95% CI: −11.88 to −3.51, *p* < 0.001) (Figure 3A). To explore potential sources of heterogeneity, we conducted meta-regression analyses considering sample size, average age, and publication year. Based on the meta-regression, sample size (*p* = 0.10, Adj. *R*^2^ = 55.31%), publication year (*p* = 0.89, Adj. *R*^2^ = -22.08%), average age (*p* = 0.58, Adj. *R*^2^ = −21.59%) had no significant effect on ALT (Figures 4A–C; Supplementary material S4A–C). Due to incomplete information, subgroup analyses were performed on treatment duration, course of disease, comorbidity and the classic treatment measures to explore possible heterogeneity sources. These subgroup analyses of course of disease, comorbidity and the classic treatment measures did not reveal any significant reduction in heterogeneity within these subgroups, suggesting that they may not be the source of heterogeneity at present (Supplementary material S5A–C). Unfortunately, due to the limited number of included articles, subgroup analysis regarding treatment duration could not be conducted (Supplementary material S5D). Therefore, more comprehensive and extensive studies are needed to investigate possible contributing factors. One hypothesis for the large heterogeneity observed is the substantial individual differences in baseline ALT levels among the included patients in these studies. Additionally, variations in detection methods and measurement biases might also have influenced the results of the heterogeneity test. To assess the robustness of the findings, sensitivity analysis was performed by sequentially removing one study at a time and re-analyzing the remaining studies. The results showed that the pooled effect sizes remained consistent, indicating the robustness of the findings (Figure 5A).

##### 3.4.1.2 DCHD vs. conventional treatment

Two studies (39, 40), involving a total of 133 patients, evaluated the efficacy of DCHD compared to conventional treatment on ALT levels. Based on the heterogeneity test (*p* < 0.01, *I*^2^ = 96%), a random effect model was finally selected for statistical analysis. The pooled effect showed no significant difference between DCHD and conventional treatment in terms of ALT levels (MD = −12.54 U/L, 95% CI: −31.58 to 6.49, *p* = 0.20) (Figure 3B). This indicated that DCHD alone may also have the same effect as conventional treatment on ALT. However, when switching to a fixed effect model, the result changed, suggesting that the robustness of the result is uncertain. Because of the small number of related studies and the substantial differences in results among them, the efficacy of DCHD alone on ALT levels cannot be determined at this time.

#### 3.4.2 AST

##### 3.4.2.1 Conventional treatment combined with DCHD vs. conventional treatment

**Figure 6:**
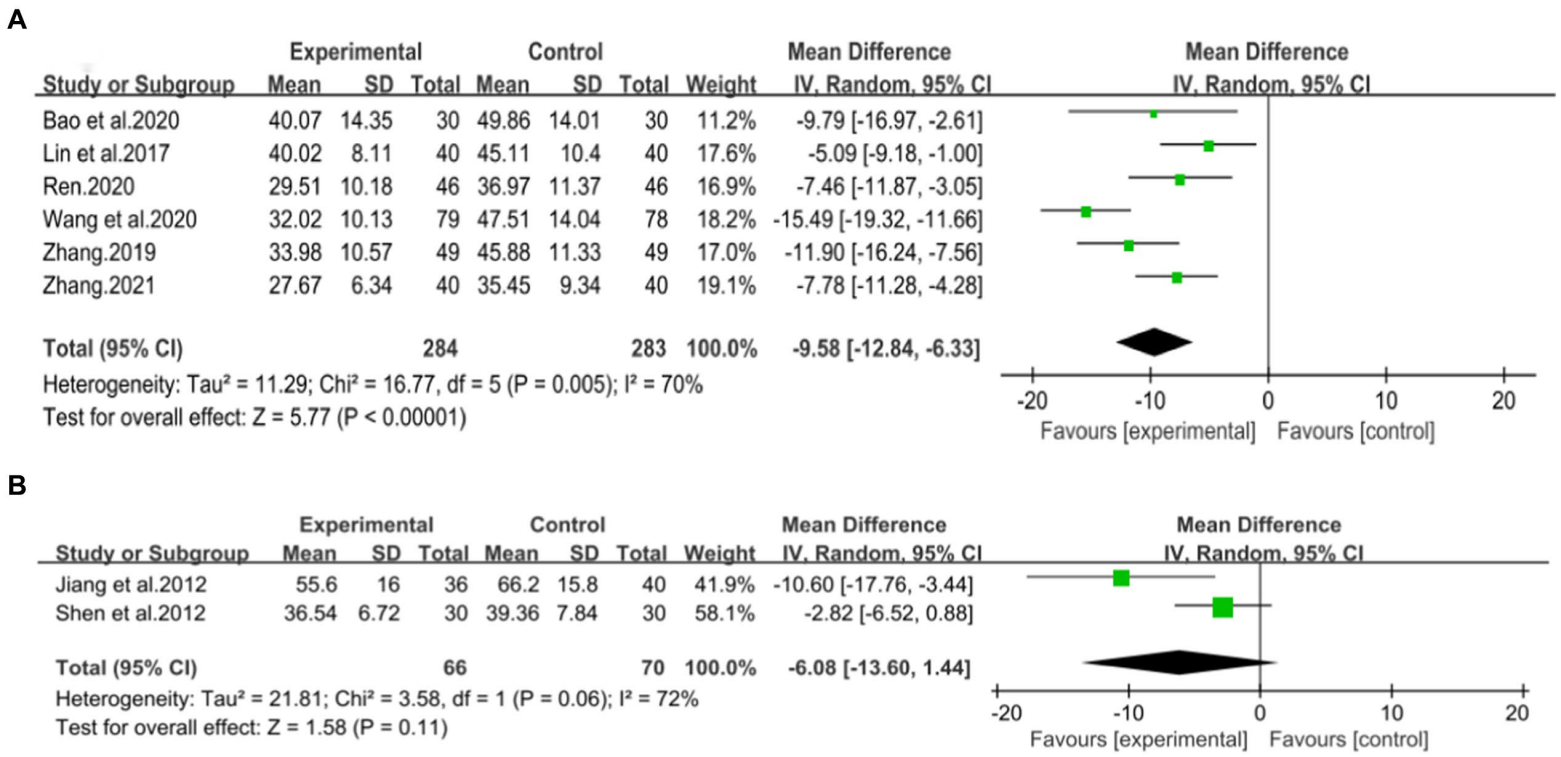
Forest plot of the AST. **(A)** DCHD combined with conventional treatment vs. conventional treatment. **(B)** DCHD vs. conventional treatment.

**Figure 7:**
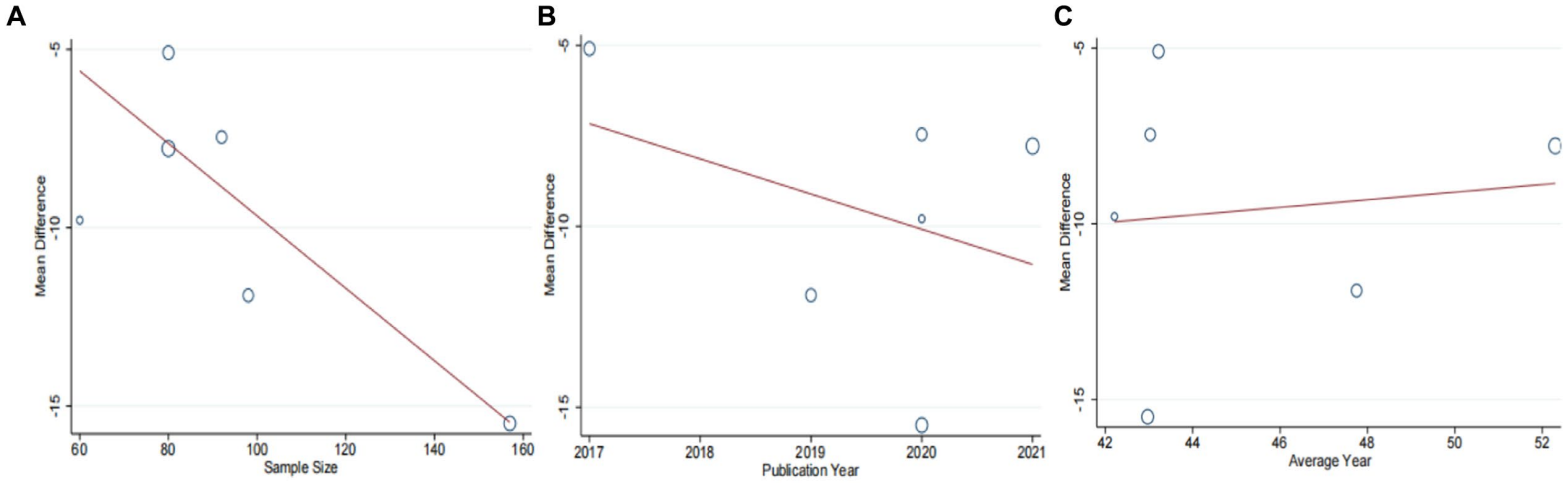
Meta-regression of the AST for DCHD combined with conventional treatment vs. conventional treatment. **(A)** Sample size. **(B)** Publication year. **(C)** Average year.

Six studies (42–47) involving 567 patients had reported on the efficacy of combining conventional treatment with DCHD compared to conventional treatment alone. Based on the heterogeneity test (*p* = 0.005, *I*^2^ = 70%), a random effect model was finally chosen for statistical analysis. The pooled effect demonstrated that the combination group had significantly lower AST levels compared to the conventional treatment group (MD = −9.58 U/L, 95% CI: −12.84 to −6.33, *p* < 0.01) (Figure 6A). Because of the high heterogeneity, meta-regressions were performed on sample size, average age, publication year and proportion of man to seek possible source. The meta-regression analysis based on sample size showed a linear relationship, and the decrease in Tau^2^ from 11.29 to 0 indicated that sample size may be the source of heterogeneity, explaining 100% of the variation between studies (*p* = 0.028, Adj. *R*^2^ = 100%) (Figure 7A; Supplementary material S6A). Analyzing the regression diagram, we could find that the decrease in AST gradually increased with sample size. This may be due to the presence of a large chance when the sample size is too small. Moreover, meta-regression according to average year (*p* = 0.58, Adj. *R*^2^ = −32.40%), publication year (*p* = 0.50, Adj. *R*^2^ = −14.88%) showed no significant effect on AST (Figures 7B,C; Supplementary material S6B,C). As some subgroups included only one study, a subgroup analysis of course of disease, comorbidity, and treatment duration could not be performed. Further analysis revealed that classic treatment measures did not entirely reduce the heterogeneity within each subgroup, suggesting that the factor may not be the sources of heterogeneity at present (Supplementary material S7). Sensitivity analysis demonstrated that the pooled statistics were consistent and the results were robust (Figure 5B).

##### 3.4.2.2 DCHD vs. conventional treatment

Two studies involving 136 patients evaluated the efficacy of DCHD compared to conventional treatment on AST levels (39, 40). Based on the heterogeneity test (*p* = 0.06, *I*^2^ = 72%), a random effect model was chosen for statistical analysis. The pooled effect showed no significant difference between DCHD and conventional treatment in terms of AST levels (MD = −6.08 U/L, 95% CI: −13.60 to 1.44, *p* = 0.20) (Figure 6B). Switching to a fixed effect model did not alter the result, indicating its robustness. However, due to the limited number of included studies and the substantial differences in results among them, the efficacy of DCHD alone on AST levels cannot be determined at this time.

#### 3.4.3 TC

##### 3.4.3.1 Conventional treatment combined with DCHD vs. conventional treatment

**Figure 8:**
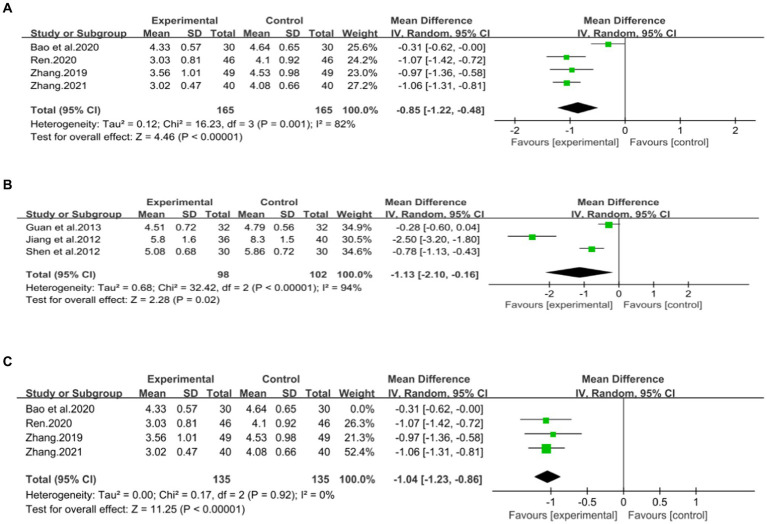
Forest plot of the TC. **(A)** DCHD combined with conventional treatment vs. conventional treatment. **(B)** DCHD vs. conventional treatment. **(C)** DCHD combined with conventional treatment vs. conventional treatment after excluding Bao et al. (44).

**Figure 9:**
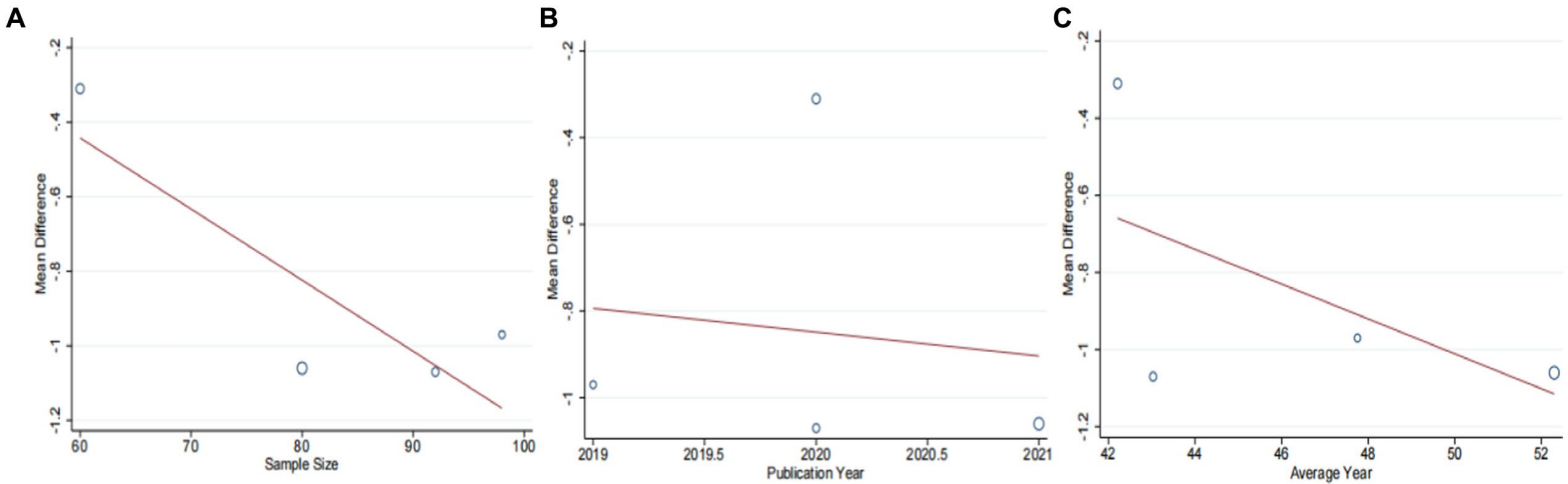
Meta-regression of the TC for DCHD combined with conventional treatment vs. conventional treatment. **(A)** Sample size. **(B)** Publication year. **(C)** Average year.

Four studies (43, 44, 46, 47), including 330 patients, investigated the effectiveness of combining conventional treatment with DCHD compared to conventional treatment alone on TC levels. The heterogeneity test (*p* = 0.001, *I*^2^ = 82%) indicated significant heterogeneity among the studies, and a random effect model was used for statistical analysis. The results showed that combining DCHD with conventional treatment significantly reduced TC levels compared to conventional treatment alone (MD = −0.85 mmol/L, 95% CI: −1.22 to 0.48, *p* < 0.01) (Figure 8A). Meta-regression analysis based on sample size, average year and publication year did not show any significant effects on TC levels (Figures 9A–C; Supplementary material S8A–C). Subgroup analysis was not possible due to the limited sample size. After excluding the study by Bao et al. (44), the mean difference changed from MD = −0.85 mmol/L to MD = −1.04 mmol/L, and heterogeneity decreased (*p* = 0.92, *I*^2^ = 0%) (Figure 8C). It showed that this study may be a course of heterogeneity. It is possible that the presence of extreme values in this article affected the overall results. Sensitivity analysis confirmed the stability of the results (Figure 5C).

##### 3.4.3.2 DCHD vs. conventional treatment

Three studies (39–41), involving 200 patients, examined the efficacy of DCHD compared to conventional treatment on TC levels. The heterogeneity test (*p* < 0.01, *I*^2^ = 94%) indicated significant heterogeneity among the studies, and a random effect model was used for statistical analysis. The results showed that DCHD significantly reduced TC levels compared to conventional treatment (MD = −1.13 mmol/L, 95% CI: −2.10 to 0.16, *p* < 0.01) (Figure 8B). Due to the limited number of studies within each subgroup, the effects of different treatment duration, courses of disease, comorbidity, and age characteristics on the results could not be clearly defined. Switching to a fixed effect model did not change the results, indicating their relative robustness (Figure 5D).

#### 3.4.4 TG

##### 3.4.4.1 Conventional treatment combined with DCHD vs. conventional treatment

**Figure 10:**
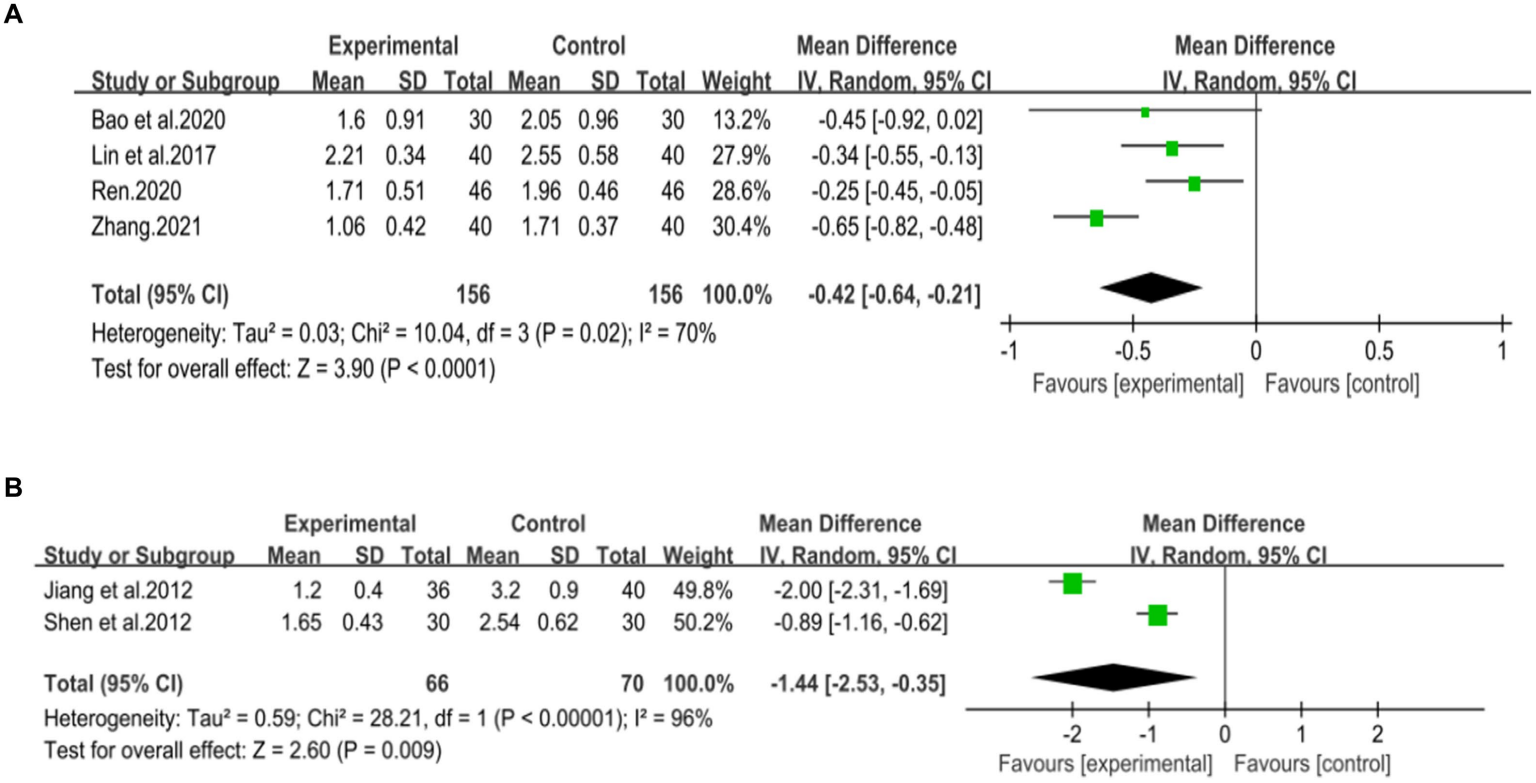
Forest plot of the TG. **(A)** DCHD combined with conventional treatment vs. conventional treatment. **(B)** DCHD vs. conventional treatment.

**Figure 11:**
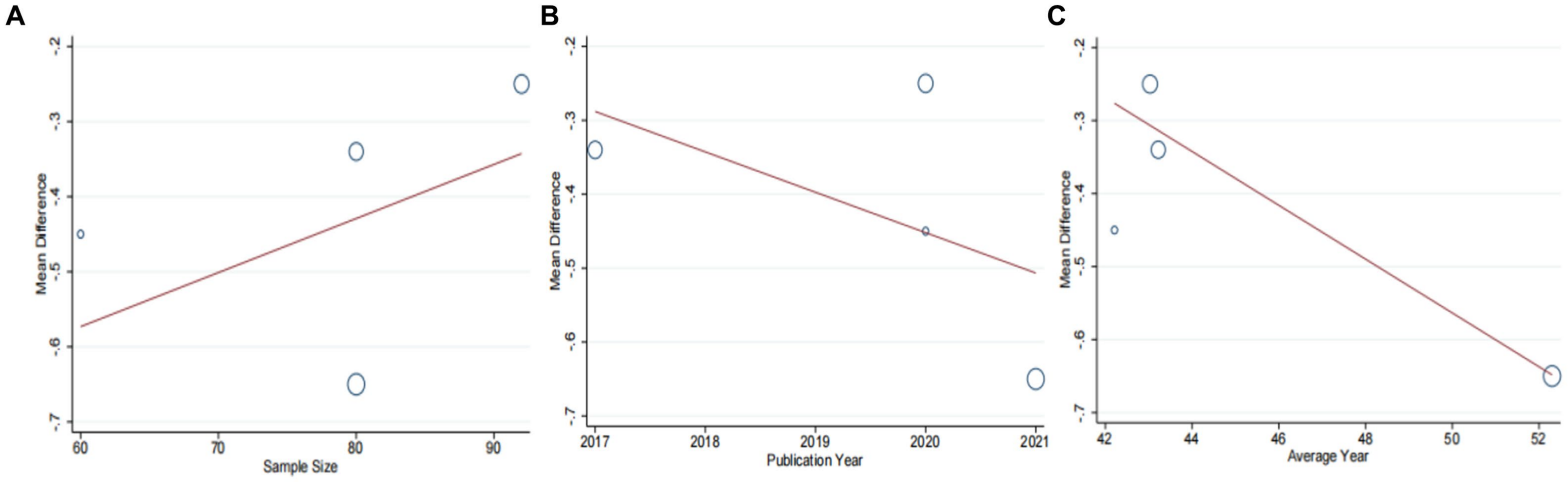
Meta-regression of the TC for DCHD combined with conventional treatment vs. conventional treatment. **(A)** Sample size. **(B)** Publication year. **(C)** Average year.

Four studies (42, 44, 46, 47), including 312 patients, investigated the effectiveness of combining conventional treatment with DCHD compared to conventional treatment alone on TG levels. The heterogeneity test (*p* = 0.02, *I*^2^ = 70%) indicated significant heterogeneity among the studies, and a random effect model was used for statistical analysis. The results showed that combining DCHD with conventional treatment reduced TG levels compared to conventional treatment alone (MD = −0.45 mmol/L, 95% CI: −0.64 to 0.21, *p* < 0.01) (Figure 10A). The meta-regression analysis accorded to average year showed a linear relationship, and the decrease in Tau^2^ from 0.227 to 0 indicated that average year may be the source of heterogeneity, explaining 100% of the variation between studies (*p* = 0.009, Adj. *R*^2^ = 100%) (Figure 11C; Supplementary material S9C). Analyzing the regression diagram, we could find that the decrease in TG gradually increased with average year. This may be due to the fact that patients of different ages have different basal levels of TG; the older the person, the higher the basal level may be, and the more pronounced the decrease in TG will be on treatment. However, because the small number of included studies, the result was still uncertain. Meta-regression analysis based on sample size (*p* = 0.16, Adj. *R*^2^ = 67.54%) and publication year (*p* = 0.51, Adj. *R*^2^ = −17.22%) did not reveal any significant effects on TG levels, suggesting that these factors were not sources of heterogeneity (Figures 11A,B; Supplementary material S9A,B). Due to the small number of studies within each subgroup, the effects of different treatment duration, courses of disease, comorbidity, and classic treatment measures on the results could not be clearly defined. Switching to a fixed effect model did not change the results, indicating their relative robustness.

##### 3.4.4.2 DCHD vs. conventional treatment

Two studies (39, 40), involving 136 patients, reported on the effectiveness of DCHD compared to conventional treatment in reducing TG levels. The heterogeneity test (*p* < 0.01, *I*^2^ = 96%) indicated significant heterogeneity among the studies, thus a random effect model was used for statistical analysis. The pooled effect showed that DCHD significantly reduced TG levels compared to conventional treatment (MD = −1.44 mmol/L, 95% CI: −2.53 to −0.35, *p* < 0.01) (Figure 10B). Due to the limited number of studies within each subgroup, subgroup analysis could not be performed.

### 3.5 Secondary outcomes

#### 3.5.1 γ-GGT

##### 3.5.1.1 Conventional treatment combined with DCHD vs. conventional treatment

**Figure 12:**
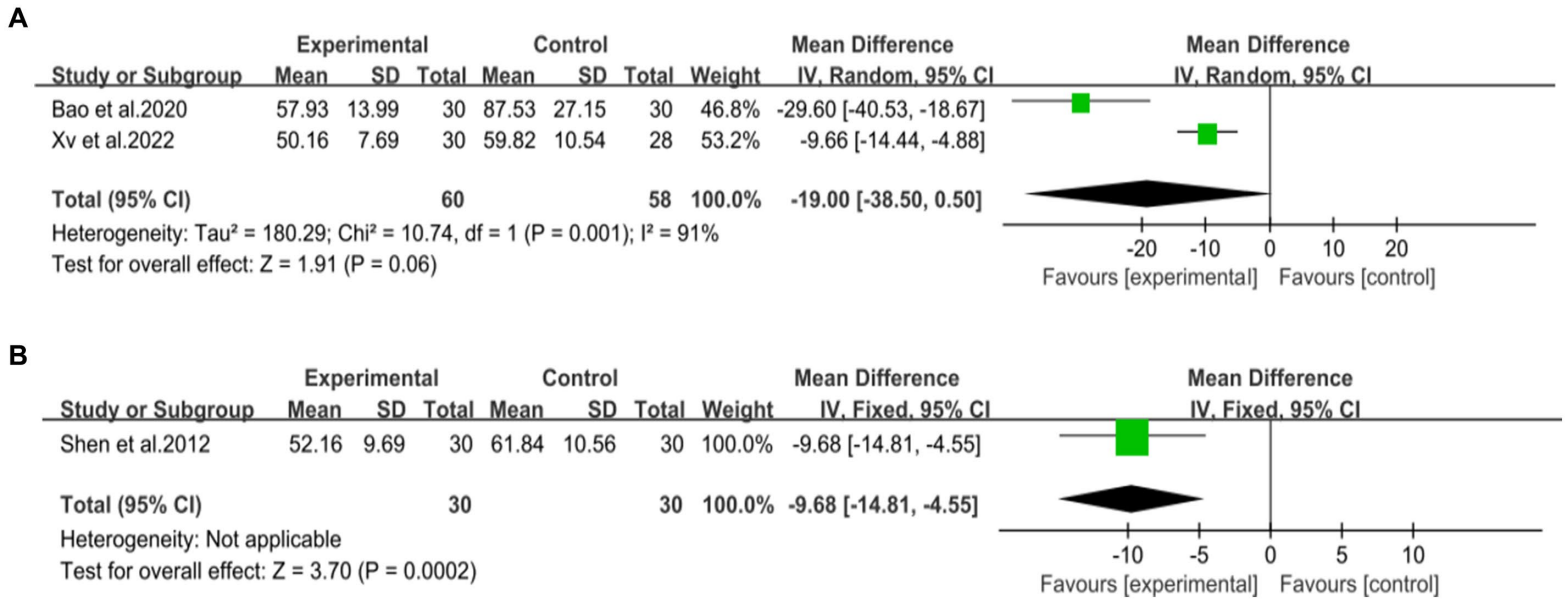
Forest plot of the γ-GGT. **(A)** DCHD combined with conventional treatment vs. conventional treatment. **(B)** DCHD vs. conventional treatment.

Two studies (44, 48), involving 118 patients, reported on the efficacy of combining DCHD with conventional treatment compared to conventional treatment alone in terms of γ-GGT levels. The heterogeneity test (*p* < 0.01, *I*^2^ = 91%) indicated significant heterogeneity among the studies, and a random effect model was applied. The pooled effect suggested no significant difference between the combination with DCHD group and the conventional treatment group (MD = −19 U/L, 95% CI: −38.5 to 0.50, *p* = 0.06) (Figure 12A). Switching to a fixed effect model did not change the result, indicating its relative robustness.

##### 3.5.1.2 DCHD vs. conventional treatment

One study (40), involving 60 patients, reported that DCHD reduced γ-GGT levels compared to conventional treatment (MD = −9.68 U/L, 95% CI: −14.81 to −4.55, *p* < 0.01) (Figure 12B).

#### 3.5.2 HDL-C

##### 3.5.2.1 Conventional treatment combined with DCHD vs. conventional treatment

**Figure 13:**
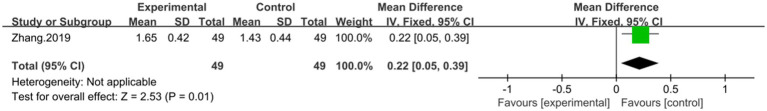
Forest plot of the HDL-C. DCHD combined with conventional treatment vs. conventional treatment.

One study (43), containing 95 patients, reported on the efficacy of combining DCHD with conventional treatment compared to conventional treatment alone in terms of HDL-C levels. The result demonstrated that combined with DCHD led to an increase in HDL-C levels (MD = 0.22 mmol/L, 95% CI: 0.05 to 0.39, *p* = 0.01) (Figure 13).

##### 3.5.2.2 DCHD vs. conventional treatment

Not reported.

#### 3.5.3 LDL-C

##### 3.5.3.1 Conventional treatment combined with DCHD vs. conventional treatment

**Figure 14:**
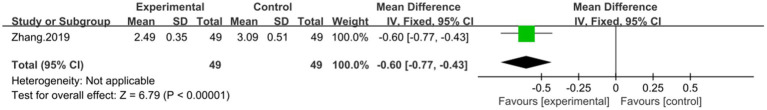
Forest plot of the LDL-C. DCHD combined with conventional treatment vs. conventional treatment.

Involving 95 patients, one study (43) reported on the efficacy of combination with DCHD compared to conventional treatment alone in terms of LDL-C levels. The result showed that combination with DCHD led to a reduction in LDL-C levels (MD = −0.60 mmol/L, 95% CI: −0.77 to −0.43, *p* < 0.01) (Figure 14).

##### 3.5.3.2 DCHD vs. conventional treatment

Not reported.

#### 3.5.4 BMI

##### 3.5.4.1 Conventional treatment combined with DCHD vs. conventional treatment

**Figure 15:**
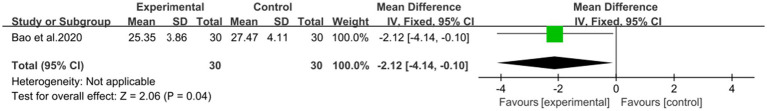
Forest plot of the BMI. DCHD combined with conventional treatment vs. conventional treatment.

Another study (44), involving 60 patients, showed that combining DCHD with conventional treatment reduced BMI levels (MD = −2.12 kg/m^2^, 95% CI: −4.14 to −0.10, *p* = 0.04) (Figure 15).

##### 3.5.4.2 DCHD vs. conventional treatment

Not reported.

### 3.6 Adverse events

Among the 10 included studies, only one study reported adverse events. Lin and Li’s study (42) reported adverse events in control group, which use polyene phosphatidylcholine alone. No significant adverse effects were seen with the combined use of DCHD in the experimental group. The results indicated that DCHD is relatively safe. However, given the limited sample size, further studies are needed to confirm its safety.

### 3.7 Publication bias

Due to the small number of trials included in each subgroup, it was not possible to adequately analyze publication bias.

### 3.8 Assessment of evidence quality

The GRADE method was used to assess the quality of evidence. The overall quality of evidence for each outcome was found to be moderate to very low due to high risk of bias, inconsistency between studies, and imprecision in results.

## 4 Discussion

### 4.1 Main results of this research

Non-alcoholic fatty liver disease is a common chronic liver disease characterized by fat accumulation in the liver, but without a history of excessive alcohol consumption, which is a disease reversibly related to obesity and diabetes mellitus (1, 17). Its pathological process mainly consists of five aspects such as fat accumulation, oxidative stress and cell injury, inflammatory response, hepatocyte apoptosis and fibrosis and cirrhosis (49). DCHD is a traditional Chinese medicine formula that has been studied in the treatment of NAFLD, showing promising results. In this article, we conducted a comprehensive search on both Chinese and English databases and analyzed the efficacy and safety of DCHD in the treatment of NAFLD. We focused on outcome indicators related to liver function, lipid metabolism, insulin resistance, and islet function. We also explored sources of heterogeneity through meta-regression and subgroup analysis, while assessing the quality of evidence. Both original DCHD and modified DCHD formulations were included in the analysis. Like other traditional Chinese medicine prescriptions, DCHD consists of specific herbs with specific functions. It is primarily used for NAFLD patients with heat stagnation in the liver and stomach syndrome. It has the function of reconciliation Shaoyang and discharging internal heat However, symptoms can vary among patients, so modifications to the original DCHD may be necessary based on accompanying symptoms and individual differences. This allows for better adaptation of the formula to each patient’s situation and enhances its effectiveness. Modified DCHD has a similar composition and therapeutic effect as the original DCHD, making it suitable for different conditions of NAFLD patients and placing it in the same category. Through comprehensive analysis, we obtained several findings. A total of 144 articles were retrieved, and 10 were included in the meta-analysis. The risk of bias assessment revealed that the methodological quality of the included studies was not high, primarily due to inadequate reporting on random sequence generation, allocation concealment, and blinding implementation.

Our main finding suggests that DCHD, either alone or in combination with conventional treatment, can significantly improve liver function and regulate lipid metabolism. The combination of DCHD with conventional treatment was found to be particularly effective in improving liver function and reducing ALT (MD = −7.69 U/L, 95% CI: −11.88 to −3.51, *p* < 0.001), AST (MD = −9.58 U/L, 95% CI: −12.84 to −6.33, *p* < 0.01) and γ-GGT (MD = −19 U/L, 95% CI: −38.5 to 0.50, *p* = 0.06) levels compared to conventional treatment alone, and it also had a certain impact on blood lipid, which could reduce the levels of TC (MD = −0.85 mmol/L, 95% CI: −1.22 to 0.48, *p* < 0.01), TG (MD = −0.45 mmol/L, 95% CI: −0.64 to 0.21, *p* < 0.01) and LDL-C (MD = −0.60 mmol/L, 95% CI: −0.77 to −0.43, *p* < 0.01) as well as improving the HDL-C level (MD = 0.22 mmol/L, 95% CI: 0.05 to 0.39, *p* = 0.01). In addition, the combined use of DCHD also could reduce the levels BMI (MD = −2.12 kg/m^2^, 95% CI: −4.14 to −0.10, *p* = 0.04). But the effect on the CT ratio of liver/spleen level was not significant (MD = 0.70, 95% CI: −0.01 to 0.15, *p* = 0.08). When DCHD was used alone, it had the similar effect as conventional treatment in reducing ALT (MD = −12.54 U/L, 95% CI: −31.58 to 6.49, *p* = 0.20) and AST (MD = −6.08 U/L, 95% CI: −13.60 to 1.44, *p* = 0.20) levels. However, it could significantly reduce the levels of TC (MD = −1.13 mmol/L, 95% CI: −2.10 to 0.16, *p* < 0.01), TG (MD = −1.44 mmol/L, 95% CI: −2.53 to −0.35, *p* < 0.01) and γ-GGT (MD = −9.68 U/L, 95% CI: −14.81 to −4.55, *p* < 0.01) compared with conventional treatment. Based on the included experiments, it is unclear whether DCHD alone could ameliorate the levels of HDL-C，LDL-C and BMI. Based on these results, it appears that combining DCHD with conventional treatment could serve as a beneficial complementary therapy for NAFLD patients.

However, it is important to note that our analysis revealed substantial heterogeneity in these findings. We conducted meta-regression and subgroup analyses to explore the potential sources of heterogeneity. Unfortunately, due to the limited number of studies within each subgroup, we were unable to identify the specific source of heterogeneity. Our analysis suggests that measurement bias resulting from the detection method may contribute to the observed heterogeneity. Additionally, methodological deficiencies in the included studies, such as the lack of blinding and allocation concealment, may also contribute to the heterogeneity. It is worth mentioning that none of the included studies claimed to have performed a placebo control, indicating insufficient evidence for comparing DCHD alone versus placebo. Of the 10 studies included in our analysis, only one study reported adverse events. However, the details of these adverse events were not provided by Lin and Li’s study (42). This suggests that researchers may not have given sufficient attention to adverse events in their studies. Through consulting relevant information, we found that the main adverse reactions of DCHD were gastrointestinal reactions, such as stomach pain, diarrhea, etc. A few physical weak people can appear dizziness, headache and other symptoms. Patients with advanced cirrhosis and cholestasis should be used with caution, which has the risk of aggravating jaundice (50). Nevertheless, no serious adverse events were observed, indicating that DCHD is relatively safe when used correctly. It is important to note that drug safety should be evaluated using multiple indicators, such as blood routine, urine routine, stool routine, liver and kidney function, electrocardiogram, and patient-reported discomfort. Therefore, more high-quality studies addressing these aspects are needed to further confirm the safety profile of DCHD. It is crucial to exercise caution when considering the clinical application of DCHD, despite the literature included in this study not indicating an increased occurrence of adverse reactions.

In TCM, Chinese Thorowax Root (Chaihu, *Bupleurum falcatum* L.), Baical Skullcap Root (Huangqin, *Scutellaria baicalensis* Georgi) and Rhubarb (Dahuang, *Rheum palmatum* L.) are cold in nature, which are included in DCHD. Excessive dosage or administration of DCHD to patients with weak constitution can potentially harm the yang qi of the spleen and stomach, leading to symptoms such as abdominal distension and diarrhea. Therefore, the clinical use should concentrate on syndrome differentiation and physical differences, which varies from person to person. It is important to adjust the dosage of each herb based on individual circumstances to minimize the occurrence of adverse reactions. Currently, there are no relevant reports of the DCHD toxicity. Pharmacological studies have shown that Pinellia Tuber (Banxia, *Pinellia ternata* (Thunb.) Makino) contains alkaloids, lectins, and toxic raphides of calcium oxalate, making it a potentially poisonous herb (50–52). These components can stimulate the mucosa and cause hepatorenal and pregnancy toxicity (33, 53–56). However, studies have found that processing Pinellia Tuber can greatly reduce the occurrence of poisoning events (57). Therefore, for safety purposes, strictly processed Pinellia Tuber should be used in DCHD. Processing destroys the structure of calcium oxalate raphides and denatures and deactivates lectin proteins, achieving a detoxification effect (58, 59). It should be noted that different processing methods may yield different effects on Pinellia Tuber (19, 57). In clinical practice, the dosage of Pinellia Tuber should be adjusted flexibly based on the patient’s condition and used in combination with other Chinese medicines like ginger to maximize therapeutic effects and reduce toxicity. Further pharmacological and toxicological studies are needed to explore the toxicity of DCHD. Due to the limited number of trials included in each subgroup (less than 10), it was not possible to adequately analyze publication bias.

### 4.2 Study on the internal possible mechanism

With the accelerating pace of our life, the incidence of NAFLD patients increases year by year and shows a trend of becoming younger. DCHD has the advantages of multi-component and multi-target, targeting all links and pathological products of NAFLD pathogenesis, and plays a unique role in preventing and controlling NAFLD. In DCHD, the main components of Chinese Thorowax Root are pentacyclic triterpenoids and volatile oil, which mainly affect the main links of liver injury process by protecting the liver cell membrane, promoting the production of liver protective factor NO, improving the SOD activity and reducing the MDA content (60). White peony root is rich in monoterpene glycosides, mainly paeonifloridin, lactonidin and paeonifloridin, mainly through the inhibition of the activity of immune cells and the function of overactivated immune cells to play the function of immune regulation; and paeoniflorin can promote the release of FFA from isolated adipose tissue, reduce the MDA content and increase the T-AOC level. The main components of Immature Orange Fruit are flavonoids, alkaloids and volatile oil, the extract of Immature Orange Fruithas have the ability to remove the superoxide anion radical and hydroxyl radical, and to inhibit the peroxidation of liver, kidney and heart tissues; at the same time, the extract could improve the hepatic antioxidant activity and inhibit lipid peroxidation in diabetic mice (61, 62). Clinical studies have confirmed that DCHD can reduce blood bile acid concentration by upregulating the expression of farnesol receptor (FXR) mRNA to reduce liver damage (63). Pharmacological studies have shown that DCHD can reduce vitreous degeneration, inhibit the production of glutamic-pyruvic transaminase (GPT), thus enhancing the activity of tryptophan oxygenase and glutamine synthetase, can inhibit carbon tetracloride, thus slowing down the development of cirrhosis, to achieve the purpose of protecting the liver (64). Studies have demonstrated that DCHD can improve glucose and lipid metabolism, enhance antioxidant enzyme activity, reduce reactive oxygen species (ROS), superoxide dismutase (SOD), and malondialdehyde (MDA) levels. It also upregulates the expression of pancreatic duodenal homeobox-1 (PDX-1) and MaFA mRNA in pancreatic tissue (19). The underlying mechanism may involve the regulation of adiponectin and leptin gene expression in adipose tissue, inhibition of adipose tissue proliferation and differentiation, and modulation of intestinal flora balance (65). Network pharmacology studies exploring the mechanism of DCHD in preventing and treating NAFLD have revealed its potential association with the tumor HIF-1 signaling pathway, tumor necrosis factor (TNF) signaling pathway, insulin resistance pathway, among others. This suggests that DCHD may regulate inflammatory responses, promote vascular endothelial cell proliferation and differentiation, and mitigate vascular endothelial cell damage caused by hyperlipidemia (66, 67).

In conclusion, the protective effects of DCHD on liver function and its role in regulating lipid metabolism, reducing insulin resistance, and improving islet cell function may be attributed to its ability to inhibit oxidative stress, modulate inflammatory responses, lower blood bile acid concentration, alleviate hyaline degeneration of liver tissue, regulate insulin signal transduction, influence the expression of adiponectin and leptin genes, and maintain a balanced intestinal flora.

### 4.3 Limitation of this study

Despite utilizing standard analytical methods, this study has several limitations that need to be acknowledged. Firstly, the methodological quality of the included studies was generally low, with unclear randomization methods and a lack of blinding and allocation concealment. The absence of placebo-controlled trials also introduces potential bias and is a major limitation of the study, therefore the claims made for DCHD’s efficacy can be fully validated. Furthermore, DCHD’s effect on clinical symptoms, hormonal imbalance, neurological impacts, safety in terms of metabolism and toxicity had not been mentioned. More and more comprehensive clinical studies are needed in the future to further validate the efficacy of DCHD in NAFLD. Secondly, all the included studies were conducted in single-center settings with small sample sizes, which may limit their representativeness and generalizability. Studies with small sample sizes are subject to greater chance, which makes the results less reliable. Thirdly, due to the limited number of articles and incomplete information, our ability to conduct comprehensive heterogeneous analysis and explore specific subgroups was restricted. Most studies did not report comorbidity, preventing a subgroup analysis on this factor. In fact, different comorbidity may have opposite effects on DCHD in treating NAFLD. Additionally, there was poor standardization in reporting treatment outcomes. Fourthly, the included studies were exclusively from Chinese literature, which may introduce ethnic and regional limitations. Furthermore, none of the included studies were registered or had study protocols available, and there may be potential publication bias as positive results are more likely to be published in China. Lastly, most studies did not report adverse events, making it difficult to assess the safety profile of DCHD in NAFLD treatment. Therefore, the efficacy of DCHD in treating NAFLD remains uncertain. More high-quality trials with large samples and including placebo control groups are needed from different countries in the future to validate the therapeutic effect of DCHD in NAFLD. In addition to this, the researchers should also pay more attention to the occurrence of adverse reactions during the treatment and record them truthfully.

### 4.4 Implications for clinical practice and future research

Based on the aforementioned findings and limitations, the following recommendations are proposed for future research and practice: First, enhance the rigor of research protocols and strengthen quality control measures. Special attention should be given to implementing center randomization, allocation concealment, and blinding. Placebo controls should be appropriately utilized to eliminate the influence of psychological factors and comprehensively evaluate the true efficacy and adverse effects of experimental drugs. Secondly, consider conducting multicenter studies with a reasonable calculation of sample size to enhance the reliability and representativeness of research results. Thirdly, adhere strictly to the Consolidated Standards of Reporting Trials (CONSORT) statement when reporting randomized controlled trials (RCTs). Particular emphasis should be placed on reporting age, disease duration, treatment duration, proportion of men, and presence of comorbidity to explore possible sources of heterogeneity and conduct further statistical analysis on the dominant population. Fourthly, ensure clinical trial registration prior to initiation and transparent reporting of both positive and negative results to reduce publication bias and ensure information transparency. Finally, pay close attention to the observation and monitoring of adverse events, establishing strict procedures for handling and reporting such events to provide effective evidence regarding the safety of DCHD.

## 5 Conclusion

In conclusion, our findings suggest that combining DCHD with conventional treatment for NAFLD offers advantages over conventional treatment alone, leading to improved liver function, regulated lipid metabolism, reduced insulin resistance, modulated insulin function, and decreased BMI. While DCHD used alone also shows potential in improving liver function and regulating lipid metabolism, there is currently insufficient evidence to determine its effects on insulin resistance, pancreatic islet function, and BMI. Additionally, DCHD appears to have a relatively safe profile. This suggests that DCHD may have a positive effect on NAFLD. However, due to the limited number of included studies, small sample sizes, and poor methodological quality, the evidence supporting these findings remains uncertain, and caution should be exercised when interpreting and applying the results. In the treatment of NAFLD, clinical decisions should still consider the overall patient situation. Moving forward, more high-quality, large-sample, multicenter, randomized, double-blind, placebo-controlled studies are needed to provide robust evidence for the clinical application of DCHD.

## Data availability statement

The original contributions presented in the study are included in the article/Supplementary material, further inquiries can be directed to the corresponding authors.

## Author contributions

ZM: Formal analysis, Methodology, Software, Supervision, Writing – original draft, Writing – review & editing. TG: Formal analysis, Investigation, Supervision, Writing – original draft. YW: Methodology, Software, Supervision, Writing – review & editing. JL: Formal analysis, Investigation, Supervision, Writing – review & editing. JY: Methodology, Supervision, Writing – review & editing. LM: Data curation, Supervision, Writing – original draft, Writing – review & editing.
