# Corrigendum: The efficacy and safety of Dachaihu decoction in the treatment of nonalcoholic fatty liver disease: a systematic review and meta-analysis

**DOI:** 10.3389/fmed.2024.1523759

**Published:** 2024-11-19

**Authors:** Zhiqing Mou, Tao Gong, Yanzuo Wu, Jun Liu, Jianhua Yu, Lichan Mao

**Affiliations:** ^1^Zhejiang Chinese Medical University, Hangzhou, China; ^2^Hangzhou Xixi Hospital, Hangzhou, China; ^3^Hangzhou Hospital of Traditional Chinese Medicine, Hangzhou, China

**Keywords:** Dachaihu decoction, nonalcoholic fatty liver disease, traditional Chinese medicine, meta-analysis, systematic review

In the published article, there was an error in affiliation [1]. Instead of “[Zhejiang University of Traditional Chinese Medicine],” it should be “[Zhejiang Chinese Medical University].”

The authors apologize for this error and state that this does not change the scientific conclusions of the article in any way. The original article has been updated.

