# Supplementary material for: The efficacy and safety of Dachaihu decoction in the treatment of nonalcoholic fatty liver disease: a systematic review and meta-analysis
Source: Front Med (Lausanne). 2024 Jul 2;11:1397900. doi: 10.3389/fmed.2024.1397900 (PMC11249752; doi:10.3389/fmed.2024.1397900)

# Supplementary Material S1. PRISMA 2020 checklist

| **Section and Topic** | **Item #** | **Checklist item** |
| --- | --- | --- |
| **TITLE** | | |
| Title | 1 | Identify the report as a systematic review. |
| **ABSTRACT** | | |
| Abstract | 2 | See the PRISMA 2020 for Abstracts checklist. |
| **INTRODUCTION** | | |
| Rationale | 3 | Describe the rationale for the review in the context of existing knowledge. |
| Objectives | 4 | Provide an explicit statement of the objective(s) or question(s) the review addresses. |
| **METHODS** | | |
| Eligibility criteria | 5 | Specify the inclusion and exclusion criteria for the review and how studies were grouped for the syntheses. |
| Information sources | 6 | Specify all databases, registers, websites, organisations, reference lists and other sources searched or consulted to identify studies. Specify the date when each source was last searched or consulted. |
| Search strategy | 7 | Present the full search strategies for all databases, registers and websites, including any filters and limits used. |
| Selection process | 8 | Specify the methods used to decide whether a study met the inclusion criteria of the review, including how many reviewers screened each record and each report retrieved, whether they worked independently, and if applicable, details of automation tools used in the process. |
| Data collection process | 9 | Specify the methods used to collect data from reports, including how many reviewers collected data from each report, whether they worked independently, any processes for obtaining or confirming data from study investigators, and if applicable, details of automation tools used in the process. |
| Data items | 10a | List and define all outcomes for which data were sought. Specify whether all results that were compatible with each outcome domain in each study were sought (e.g. for all measures, time points, analyses), and if not, the methods used to decide which results to collect. |
|  | 10b | List and define all other variables for which data were sought (e.g. participant and intervention characteristics, funding sources). Describe any assumptions made about any missing or unclear information. |
| Study risk of bias assessment | 11 | Specify the methods used to assess risk of bias in the included studies, including details of the tool(s) used, how many reviewers assessed each study and whether they worked independently, and if applicable, details of automation tools used in the process. |
| Effect measures | 12 | Specify for each outcome the effect measure(s) (e.g. risk ratio, mean difference) used in the synthesis or presentation of results. |
| Synthesis methods | 13a | Describe the processes used to decide which studies were eligible for each synthesis (e.g. tabulating the study intervention characteristics and comparing against the planned groups for each synthesis (item #5)). |
|  | 13b | Describe any methods required to prepare the data for presentation or synthesis, such as handling of missing summary statistics, or data conversions. |
|  | 13c | Describe any methods used to tabulate or visually display results of individual studies and syntheses. |
|  | 13d | Describe any methods used to synthesize results and provide a rationale for the choice(s). If meta-analysis was performed, describe the model(s), method(s) to identify the presence and extent of statistical heterogeneity, and software package(s) used. |
|  | 13e | Describe any methods used to explore possible causes of heterogeneity among study results (e.g. subgroup analysis, meta-regression). |
|  | 13f | Describe any sensitivity analyses conducted to assess robustness of the synthesized results. |
| Reporting bias assessment | 14 | Describe any methods used to assess risk of bias due to missing results in a synthesis (arising from reporting biases). |
| Certainty assessment | 15 | Describe any methods used to assess certainty (or confidence) in the body of evidence for an outcome. |
| **RESULTS** | | |
| Study selection | 16a | Describe the results of the search and selection process, from the number of records identified in the search to the number of studies included in the review, ideally using a flow diagram. |
|  | 16b | Cite studies that might appear to meet the inclusion criteria, but which were excluded, and explain why they were excluded. |
| Study characteristics | 17 | Cite each included study and present its characteristics. |
| Risk of bias in studies | 18 | Present assessments of risk of bias for each included study. |
| Results of individual studies | 19 | For all outcomes, present, for each study: (a) summary statistics for each group (where appropriate) and (b) an effect estimate and its precision (e.g. confidence/credible interval), ideally using structured tables or plots. |
| Results of syntheses | 20a | For each synthesis, briefly summarise the characteristics and risk of bias among contributing studies. |
|  | 20b | Present results of all statistical syntheses conducted. If meta-analysis was done, present for each the summary estimate and its precision (e.g. confidence/credible interval) and measures of statistical heterogeneity. If comparing groups, describe the direction of the effect. |
|  | 20c | Present results of all investigations of possible causes of heterogeneity among study results. |
|  | 20d | Present results of all sensitivity analyses conducted to assess the robustness of the synthesized results. |
| Reporting biases | 21 | Present assessments of risk of bias due to missing results (arising from reporting biases) for each synthesis assessed. |
| Certainty of evidence | 22 | Present assessments of certainty (or confidence) in the body of evidence for each outcome assessed. |
| **DISCUSSION** | | |
| Discussion | 23a | Provide a general interpretation of the results in the context of other evidence. |
|  | 23b | Discuss any limitations of the evidence included in the review. |
|  | 23c | Discuss any limitations of the review processes used. |
|  | 23d | Discuss implications of the results for practice, policy, and future research. |
| **OTHER INFORMATION** | | |
| Registration and protocol | 24a | Provide registration information for the review, including register name and registration number, or state that the review was not registered. |
|  | 24b | Indicate where the review protocol can be accessed, or state that a protocol was not prepared. |
|  | 24c | Describe and explain any amendments to information provided at registration or in the protocol. |
| Support | 25 | Describe sources of financial or non-financial support for the review, and the role of the funders or sponsors in the review. |
| Competing interests | 26 | Declare any competing interests of review authors. |
| Availability of data, code and other materials | 27 | Report which of the following are publicly available and where they can be found: template data collection forms; data extracted from included studies; data used for all analyses; analytic code; any other materials used in the review. |

*From:* Page MJ, McKenzie JE, Bossuyt PM, Boutron I, Hoffmann TC, Mulrow CD, et al. The PRISMA 2020 statement: an updated guideline for reporting systematic reviews. BMJ 2021;372:n71. doi: 10.1136/bmj.n71 For more information, visit: http://www.prisma-statement.org/

**Supplementary Material S2.** search terms

(1)Non alcoholic Fatty Liver Disease;NAFLD;Nonalcoholic Fatty Liver Disease;Fatty Liver, Nonalcoholic; Fatty Livers, Nonalcoholic;Liver, Nonalcoholic Fatty;Livers, Nonalcoholic Fatty; Nonalcoholic Fatty Liver; Nonalcoholic Fatty Livers;Nonalcoholic Steatohepatitis; Nonalcoholic Steatohepatitides;Steatohepatitides, Nonalcoholic;Steatohepatitis, Nonalcoholic.

(2) Daisaikoto;Dachaihu；da chaihu；dachaihu tang；da chaihu tang; dachaihu decoction；da chaihu decoction；major bupleurum decoction; major bupleurum tang.

**Supplementary Material S3**. Components of DCHD or its modified used in the included studies

| **Reference** | **Formula** | **Components** |
| --- | --- | --- |
| Jianget al.(2012) | DCHD | Chinese Thorowax Root (Chaihu, Bupleurum falcatum L.), Baical Skullcap Root (Huangqin, Scutellaria baicalensis Georgi), Pinellia Tuber [Banxia, Pinellia ternata (Thunb.) Makino], Immature Orange Fruit (Zhishi, Citrus aurantium L.), Rhubarb (Dahuang, Rheum palmatum L.), White Paeony Root (Baishao, Paeonia lactiflora Pall.),Fresh Ginger (Shengjiang, Zingiber officinale Roscoe ,Chinese Date (Dazao, Ziziphus jujuba Mill.).(Specific dosage is unknown) |
| Shen et al.(2012) | modified DCHD | Chinese Thorowax Root (Chaihu, Bupleurum falcatum L.) 12g, Baical Skullcap Root (Huangqin, Scutellaria baicalensis Georgi) 9g, Pinellia Tuber [Banxia, Pinellia ternata (Thunb.) Makino] 9g, Immature Orange Fruit (Zhishi, Citrus aurantium L.) 9g, Rhubarb (Dahuang, Rheum palmatum L.) 6g,Red Peony Root (Chishao, Paeonia lactiflora Pall.) 9g, Fresh Ginger (Shengjiang, Zingiber officinale Roscoe) 6g,Chinese Date (Dazao, Ziziphus jujuba Mill.) 15g, Hawthorn Fruit (Shanzha, Crataegus pinnatifida Bunge) 15g, Indian Buead (Fuling,Poria cocos (Schw.) Wolf.) 15g ,Tangerine Peel(Chenpi,Citrus reticulata Blanco.) 10g,Medicated Leaven (Shenqu,Massa medicata fermentata) 10g,Ergot(Lianqiao,Forsythia suspensa.)10g,Semen Raphani (Laifuzi, Raphanus sativus L.) 10g. |
| Guan et al.(2013) | modified DCHD | Chinese Thorowax Root (Chaihu, Bupleurum falcatum L.), Baical Skullcap Root (Huangqin, Scutellaria baicalensis Georgi),Pinellia Tuber [Banxia, Pinellia ternata (Thunb.) Makino], Immature Orange Fruit (Zhishi, Citrus aurantium L.), Rhubarb (Dahuang, Rheum palmatum L.), White Paeony Root (Baishao, Paeonia lactiflora Pall.),Chinese Gentian (Longdancao, Gentianascabra Bunge), Hawthorn Fruit (Shanzha, Crataegus pinnatifida Bunge),Virgate Wormwood Herb (Yinchen,Artemisia capillaris Thunb.),Oriental Water Plantain Rhizome (Zexie, Alisma plantago-aquatica L.), Danshen Root(Danshen, Salvia miltiorrhiza Bunge). (Conventional dosage, details not available) |
| Lin et al.(2017) | modified DCHD | Chinese Thorowax Root (Chaihu, Bupleurum falcatum L.)12g, Baical Skullcap Root (Huangqin, Scutellaria baicalensis Georgi)10g, Pinellia Tuber [Banxia, Pinellia ternata (Thunb.) Makino]10g, Rhubarb (Dahuang, Rheum palmatum L.)8g,White Paeony Root (Baishao, Paeonia lactiflora Pall.)10g,Submature Bitter Orange(Zhiqiao,Citrus aurantium L.)10g,Liquorice Root (Gancao, Glycyrrhiza glabra L.)6g,Fresh Ginger (Shengjiang, Zingiber officinale Roscoe) 10g.If abdominal distension was Severe,Tangerine Peel(Chenpi,Citri Reticulatae Pericarpium)10g and Costusroot(Muxiang,Aucklandia lappa Decne.)10g were added.If deficiency of qi was obvious, Tangshen(Dangshen, Codonopsis pilosula (Franch.) Nannf.)10g were added.If deformed stool was identifified,Largehead Atractylodes Rh (Baizhu, Atractylodes macrocephala Koidz.) 10g, Indian Buead Tuckahoe(Fuling,Poria cocos (Schw.) Wolf)10g were added,Rhubarb (Dahuang, Rheum palmatum L.) was removed. |
| Zhang.(2019) | modified DCHD | Chinese Thorowax Root (Chaihu, Bupleurum falcatum L.) 15g, Baical Skullcap Root (Huangqin, Scutellaria baicalensis Georgi) 9g, Pinellia Tuber [Banxia, Pinellia ternata (Thunb.) Makino] 9g, Immature Orange Fruit (Zhishi, Citrus aurantium L.)9g, Rhubarb (Dahuang, Rheum palmatum L.) 6g,White Paeony Root (Baishao, Paeonia lactiflora Pall.) 9g,Fresh Ginger (Shengjiang, Zingiber officinale Roscoe) 15g,Chinese Date (Dazao, Ziziphus jujuba Mill.) 4 pieces,Liquorice Root (Gancao, Glycyrrhiza glabra L.) 6g.If shortness of breath was identifified,Tangshen (Dangshen, Codonopsis pilosula (Franch.) Nannf.) 10g were added.If abdominal distention was identifified,Tangerine Peel(Chenpi,Citri Reticulatae Pericarpium)10g and Costusroot(Muxiang, Aucklandia lappa Decne.)10g were added.If deformed stool was identifified, Largehead Atractylodes Rh (Baizhu, Atractylodes macrocephala Koidz.) 10g,Indian Buead Tuckahoe (Fuling,Poria cocos (Schw.) Wolf)10g were added,Rhubarb (Dahuang, Rheum palmatum L.) was removed. |
| Bao et al.(2020) | DCHD | Chinese Thorowax Root (Chaihu, Bupleurum falcatum L.) 15g, Baical Skullcap Root (Huangqin, Scutellaria baicalensis Georgi) 10g, Pinellia Tuber [Banxia, Pinellia ternata (Thunb.) Makino] 10g, Immature Orange Fruit (Zhishi, Citrus aurantium L.) 10g, Rhubarb (Dahuang, Rheum palmatum L.) 6g,White Paeony Root (Baishao, Paeonia lactiflora Pall.) 10g,Fresh Ginger (Shengjiang, Zingiber officinale Roscoe) 10g,Chinese Date (Dazao, Ziziphus jujuba Mill.)4 pieces. |
| Wang et al.(2020) | modified DCHD | Chinese Thorowax Root (Chaihu, Bupleurum falcatum L.) 12g, Baical Skullcap Root (Huangqin, Scutellaria baicalensis Georgi) 8g, Pinellia Tuber [Banxia, Pinellia ternata (Thunb.) Makino] 8g, Immature Orange Fruit (Zhishi, Citrus aurantium L.) 8g, Rhubarb (Dahuang, Rheum palmatum L.) 8g,White Paeony Root (Baishao, Paeonia lactiflora Pall.) 12g,Fresh Ginger (Shengjiang, Zingiber officinale Roscoe)10g, Sickle Senna Seed(Juemingzi,Cassia obtusifolia L.) 20g. |
| Ren.(2020) | modified DCHD | Chinese Thorowax Root (Chaihu, Bupleurum falcatum L.) 12g, Pinellia Tuber [Banxia, Pinellia ternata (Thunb.) Makino] 8g, Immature Orange Fruit (Zhishi, Citrus aurantium L.) 8g, Rhubarb (Dahuang, Rheum palmatum L.) 8g,White Paeony Root (Baishao, Paeonia lactiflora Pall.) 12g, Fresh Ginger (Shengjiang, Zingiber officinale Roscoe) 3 pices,Sickle Senna Seed(Juemingzi, Cassia obtusifolia L.) 20g.Indian Buead Tuckahoe(Fuling,Poria cocos (Schw.) Wolf) 10g, Tangerine Peel(Chenpi,Citri Reticulatae Pericarpium) 12g,Nutgrass Galingale Rhizome（Xiangfu,Cyperus rotundus L.) 12g,Hawthorn Fruit (Shanzha, Crataegus pinnatifida Bunge) 20g,Milkvetch Root (Huangqi, Astragalus mongholicus Bunge) 8g,Chinese Date (Dazao, Ziziphus jujuba Mill.) 6 pieces.If blood stasis was diagnosed,Peach Seed (Taoren, Prunus persica (L.) Batsch) 10g,Danshen Root(Danshen, Salvia miltiorrhiza Bunge) 20g,Yanhusuo Tuber (Yanhusuo,Corydalis yanhusuo W. T.Wang) 10g,Safflower(Honghua,Carthamus tinctorius L.)10g were added.If depression and stagnation of liver qi were diagnosed,Turmeric Root Tuber (Yujin, Curcuma longa L.) 12g,Finger Citron Fruit(Foshou, Citrus medica L. var. sarcodactylis Swingle.)8g, Rose(Meiguihua, Rosa rugosa Thunb.) 8g were added.If shortness of breath and blood was identifified,Tangshen(Dangshen, Codonopsis pilosula (Franch.) Nannf.) 16g, Milkvetch Root (Huangqi, Astragalus mongholicus Bunge) 24g and Largehead Atractylodes Rh (Baizhu, Atractylodes macrocephala Koidz.) 12g were added.If phlegm-dampness encumbering the spleen was diagnosed,Officinal Magnolia Bark(Houpo,Magnolia officinalis Rehd. et Wils) 10g,Villous Amomum Fruit(Sharen,Amomum villosum Lour.) 8g,Fortune’s Eupatorium Herb(Peilan,Eupatorium fortunei Turcz.) 8g,Swordlike Atracylodes Rhizome(Cangzhu, Atractylodes lancea (Thunb.) DC.) 10g were added.If damp-heat blocking in the body was diagnosed, Capillary Wormwood Herb(Yinchen,Artemisia capillaris Thunb.) 20g,Giant Knotweed Root（Huzhang,Polygonum cuspidatum Sieb. et Zucc.)20g,Stringy Stonecrop Herb (Chuipencao,Sedum sarmentosum Bge.) 20g were added. |
| Zhang.(2021) | modified DCHD | Chinese Thorowax Root (Chaihu, Bupleurum falcatum L.) 12g, Pinellia Tuber [Banxia, Pinellia ternata (Thunb.) Makino] 8g, Immature Orange Fruit (Zhishi, Citrus aurantium L.)8g, Rhubarb (Dahuang, Rheum palmatum L.) 8g,White Paeony Root (Baishao, Paeonia lactiflora Pall.) 12g, Fresh Ginger (Shengjiang, Zingiber officinale Roscoe) 3 pices,Sickle Senna Seed(Juemingzi, Cassia obtusifolia L.) 20g,Indian Buead Tuckahoe(Fuling,Poria cocos (Schw.) Wolf) 10g, Tangerine Peel(Chenpi,Citri Reticulatae Pericarpium) 12g,Hawthorn Fruit (Shanzha, Crataegus pinnatifida Bunge) 20g,Milkvetch Root (Huangqi, Astragalus mongholicus Bunge) 8g,Chinese Date (Dazao, Ziziphus jujuba Mill.) 6 pieces.If blood stasis was diagnosed,Peach Seed (Taoren, Prunus persica (L.) Batsch)10g,Danshen Root(Danshen, Salvia miltiorrhiza Bunge) 20g , Yanhusuo Tuber(Yanhusuo,Corydalis yanhusuo W. T.Wang) 10g,Safflower(Honghua,Carthamus tinctorius L.)10g were added.If depression and stagnation of liver qi were diagnosed,Turmeric Root Tuber (Yujin, Curcuma longa L.)12g,Finger Citron Fruit(Foshou, Citrus medica L. var. sarcodactylis Swingle.) 8g, Rose(Meiguihua, Rosa rugosa Thunb.)8g were added.If shortness of breath and blood was identifified,Tangshen(Dangshen, Codonopsis pilosula (Franch.) Nannf.) 16g,Milkvetch Root (Huangqi, Astragalus mongholicus Bunge) 24g and Largehead Atractylodes Rh (Baizhu, Atractylodes macrocephala Koidz.) 12g were added.If phlegm-dampness encumbering the spleen was diagnosed,Officinal Magnolia Bark(Houpo,Magnolia officinalis Rehd. et Wils) 10g,Villous Amomum Fruit(Sharen,Amomum villosum Lour.) 8g,Fortune’s Eupatorium Herb(Peilan,Eupatorium fortunei Turcz.) 8g,Swordlike Atracylodes Rhizome (Cangzhu,Atractylodes lancea (Thunb.) DC.) 10g were added.If damp-heat blocking in the body was diagnosed, Capillary Wormwood Herb(Yinchen,Artemisia capillaris Thunb.) 20g,Giant Knotweed Root（Huzhang,Polygonum cuspidatum Sieb. et Zucc.) 20g, Stringy Stonecrop Herb (Chuipencao,Sedum sarmentosum Bge.)20g were added. |
| Xv et al.(2022) | modified DCHD | Chinese Thorowax Root (Chaihu, Bupleurum falcatum L.) 10g, Pinellia Tuber [Banxia, Pinellia ternata (Thunb.) Makino] 10g, Immature Orange Fruit (Zhishi, Citrus aurantium L.) 10g, Rhubarb (Dahuang, Rheum palmatum L.) 2g,White Paeony Root (Baishao, Paeonia lactiflora Pall.) 10g,Fresh Ginger (Shengjiang, Zingiber officinale Roscoe) 10g,Baical Skullcap Root (Huangqin, Scutellaria baicalensis Georgi) 10g,Red Peony Root (Chishao, Paeonia lactiflora Pall.) 15g,Chinese Date (Dazao, Ziziphus jujuba Mill.) 5g, Milkvetch Root (Huangqi, Astragalus mongholicus Bunge)15g, Cassiabarktree Twig(Guizhi,Cinnamomum cassia Presl)10g. |

**Supplementary Material S4.**Meta-regression of the ALT for DCHD combined with conventional treatment vs. conventional treatment: (A) Sample size; (B)Publication year;(C)Average year.

A B


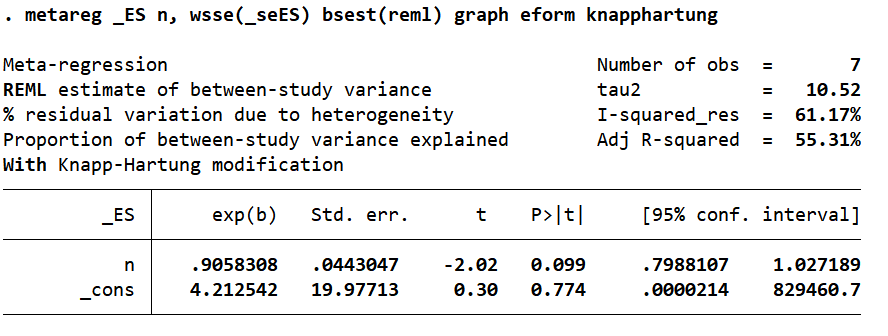

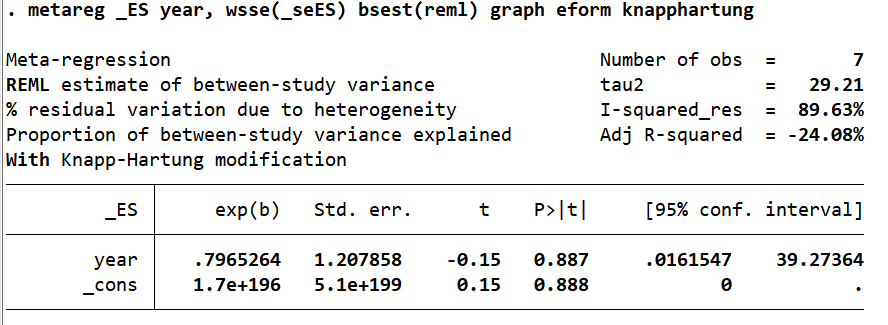


C


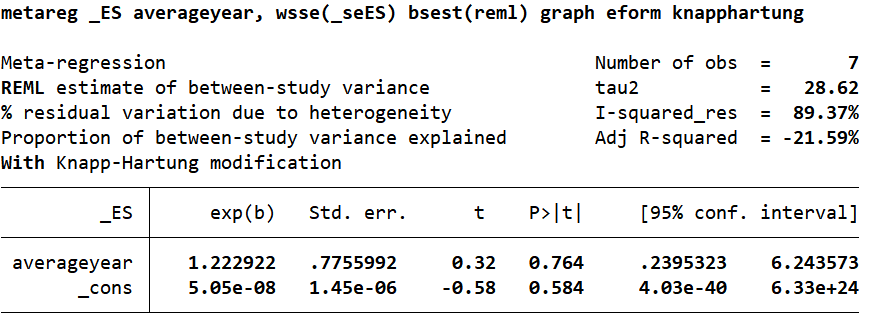


**Supplementary Material S5**. Subgroup analysis of ALT for DCHD combined with conventional treatment vs. conventional treatment: (A)course of disease;（B）comorbidity;（C）the classic treatment measures;(D) treatment duration.

A B
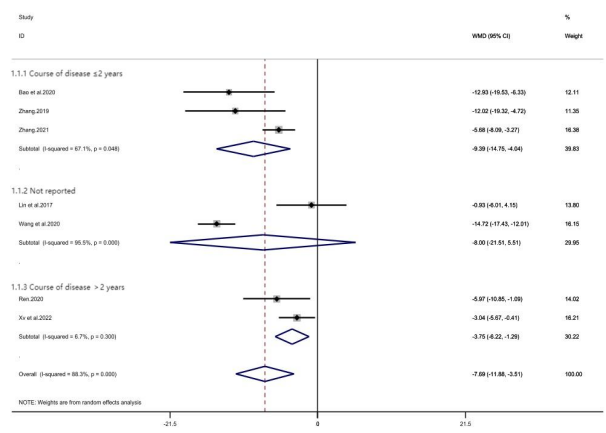

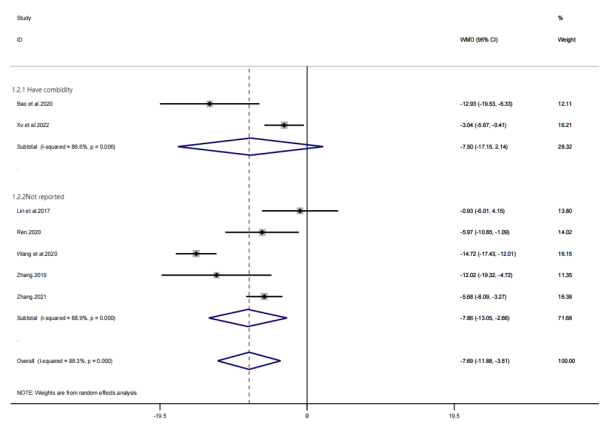


C D


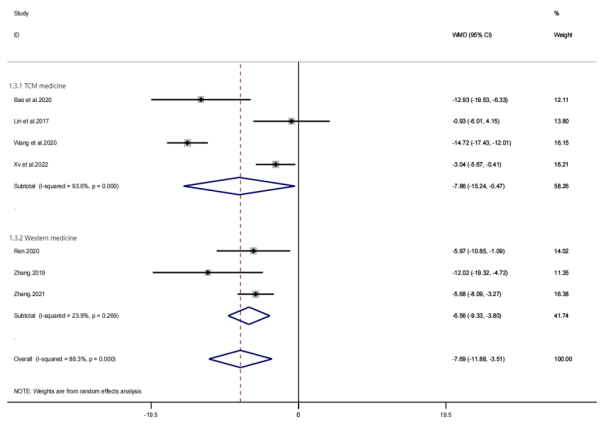

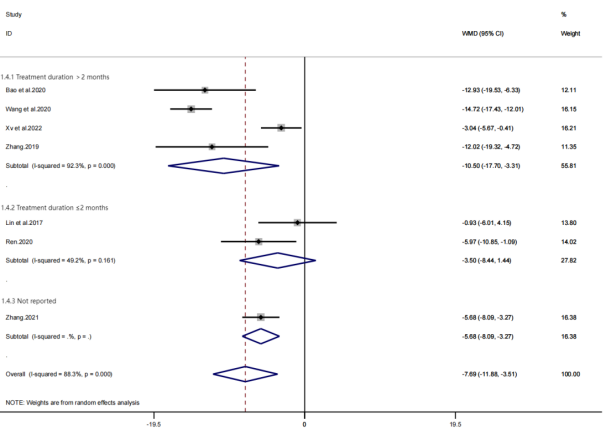


**Supplementary Material S6.**Meta-regression of the AST for DCHD combined with conventional treatment vs. conventional treatment: (A) Sample size; (B)Publication year; (C) Average year.

A B


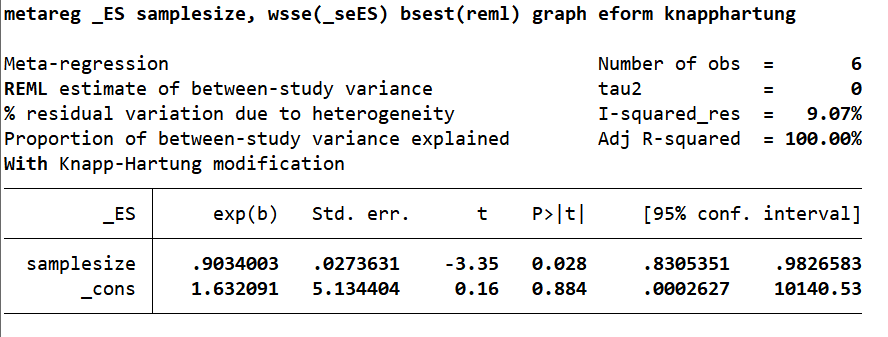

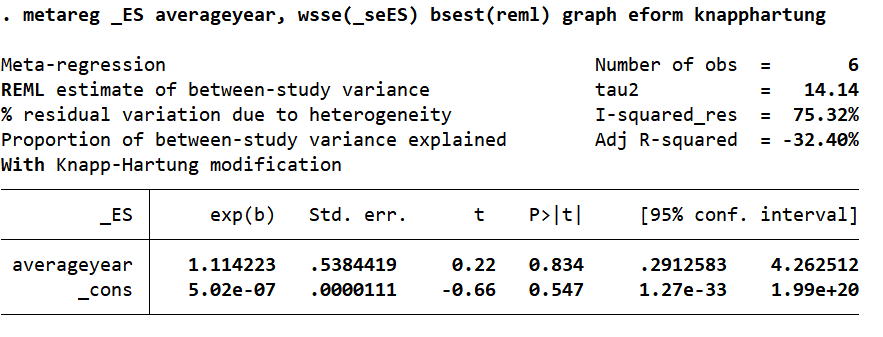


C


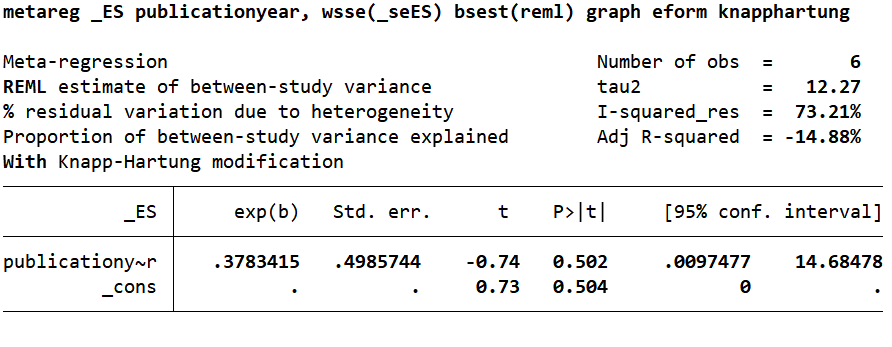


**Supplementary Material S7**. Subgroup analysis of AST for DCHD combined with conventional treatment vs. conventional treatment: the classic treatment measures.


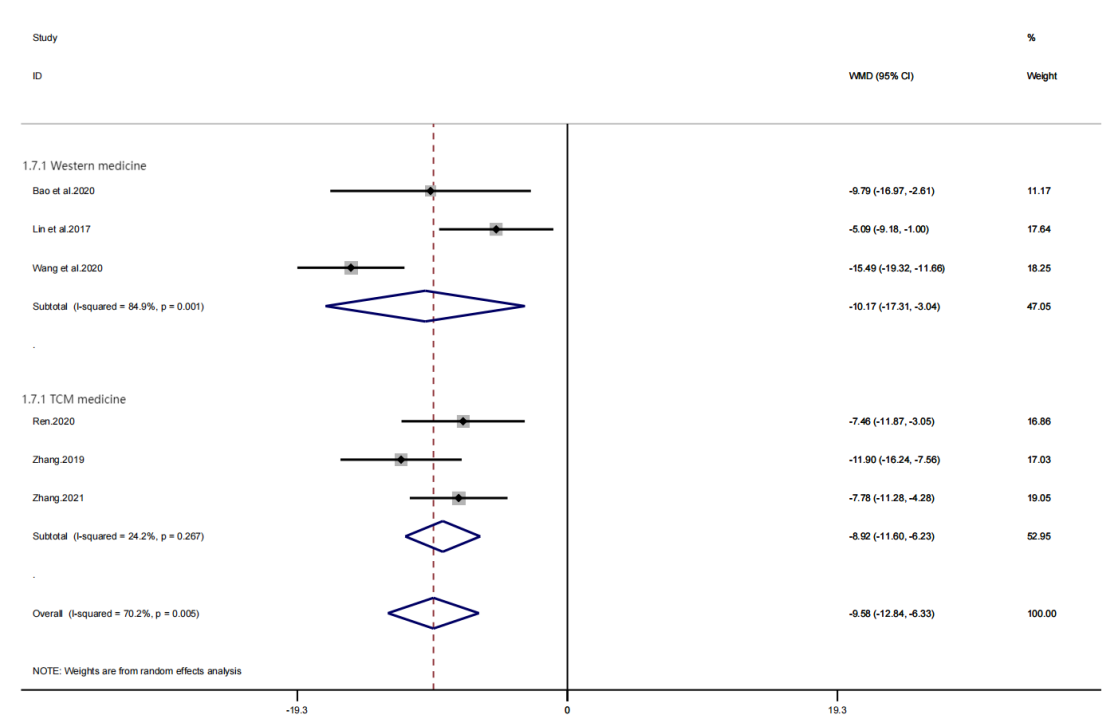


**Supplementary Material S8.**Meta-regression of the TC for DCHD combined with conventional treatment vs. conventional treatment: (A) Sample size; (B)Publication year;(C)Average year.

A B


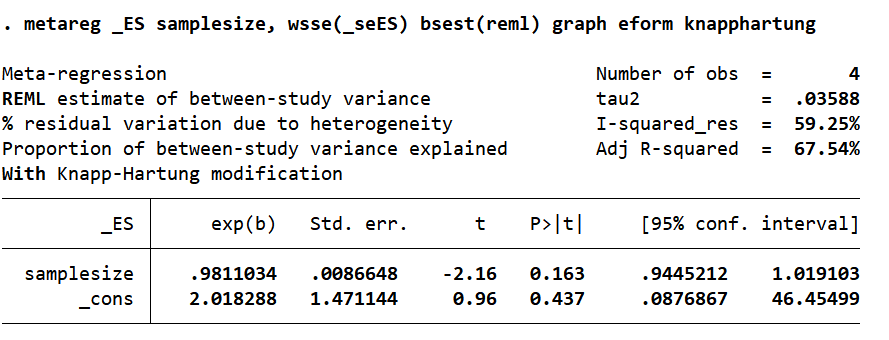

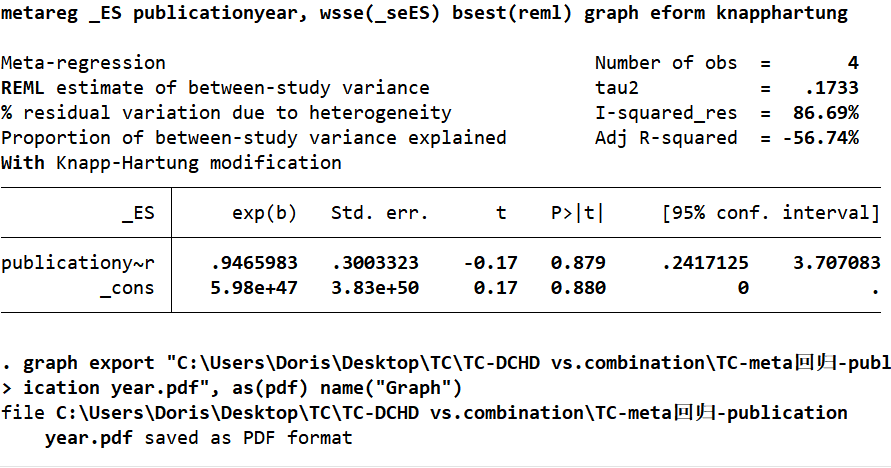


C


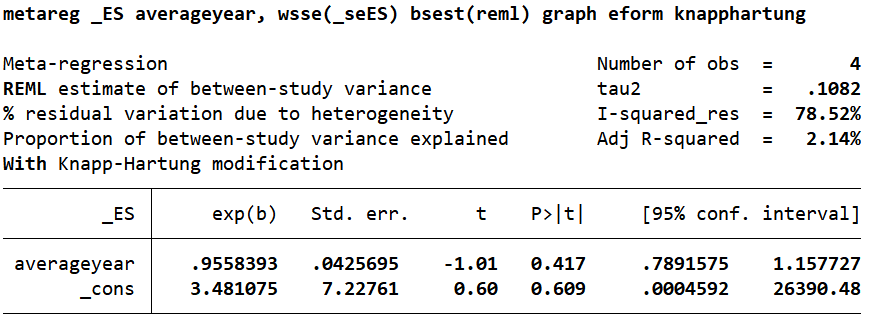


**Supplementary Material S9.**Meta-regression of the TG for DCHD combined with conventional treatment vs. conventional treatment: (A) Sample size; (B)Publication year;(C)Average year.

A B


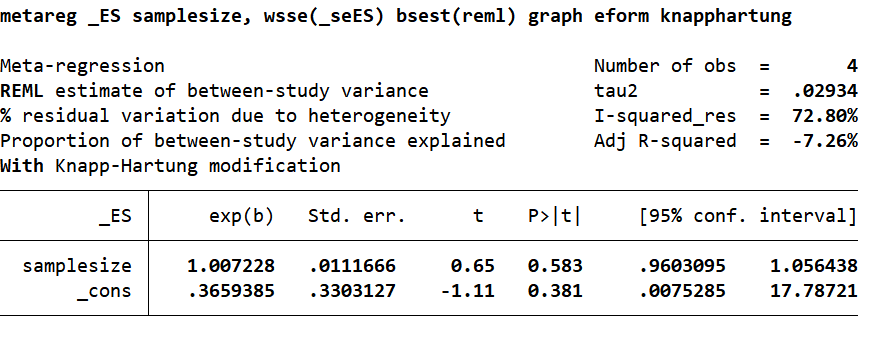

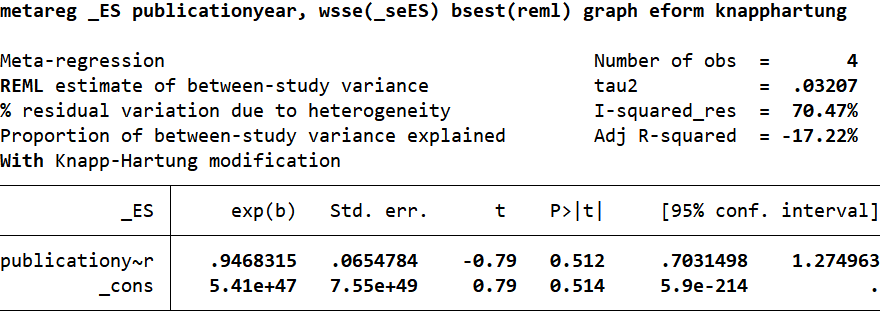


C


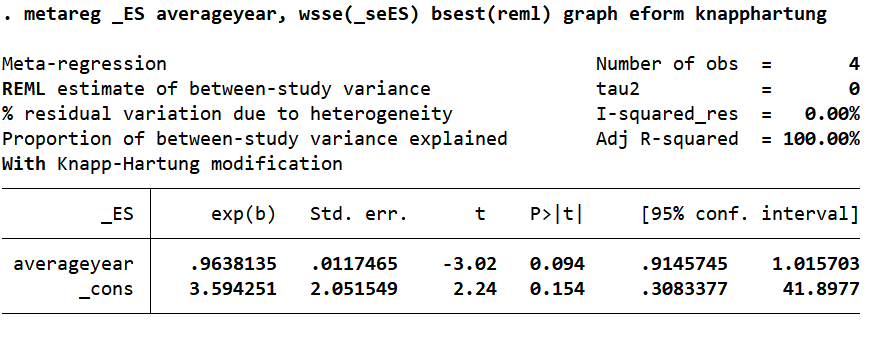

Supplement: Supplementary file 1 [file Table_1.docx]
